# Tanshinone IIA affects the malignant growth of Cholangiocarcinoma cells by inhibiting the PI3K-Akt-mTOR pathway

**DOI:** 10.1038/s41598-021-98948-z

**Published:** 2021-09-29

**Authors:** Huayuan Liu, Caiyun Liu, Mengya Wang, Dongxu Sun, Pengcheng Zhu, Ping Zhang, Xueying Tan, Guangjun Shi

**Affiliations:** 1 Department of Medicine, Qingdao University, Qingdao, China; 2 Department of Hepatobiliary Surgery, The Affiliated Qingdao Municipal Hospital of Qingdao University, Qingdao, China; 3 Department of Physiology, School of Basic Medicine, Qingdao University, Qingdao, China; 4 Graduate School of Dalian Medical University, Dalian, China; 5 Department of Gynecology, The Affiliated Qingdao Municipal Hospital of Qingdao University, Qingdao, China

**Keywords:** Cancer, Cancer therapy, Drug development

## Abstract

In the present study, we aimed to find the target of Tanshinone IIA (Tan-IIA) in Cholangiocarcinoma by network pharmacology-based prediction and investigate the possible mechanism through experimental verification. In this study, we combined Tan-IIA-specific and Cholangiocarcinoma-specific targets with protein–protein interactions (PPI) to construct a Tan-IIA targets-Cholangiocarcinoma network, and network pharmacology approach was applied to identify potential targets and mechanisms of Tan-IIA in the treatment of Cholangiocarcinoma. The anti-cancer effects of Tan-IIA were investigated by using subcutaneous tumorigenic model in nude mice and in the human Cholangiocarcinoma cell lines in vitro. Our results showed that Tan-IIA treatment considerably suppressed the proliferation and migration of Cholangiocarcinoma cells while inducing apoptosis of Cholangiocarcinoma cells. Western blot results demonstrated that the expression of PI3K, p-Akt, p-mTOR, and mTOR were inhibited by Tan-IIA. Meanwhile, After treatment with Tan-IIA, the level of Bcl2 was downregulated and cleaved caspase-3 expression increased. Further studies revealed that the anticancer effects of Tan-IIA were severely mitigated by pretreatment with a PI3K agonist. Our research provides a new anticancer strategy and strengthens support for the use of Tan-IIA as an anticancer drug for the treatment of CCA.

## Introduction

Cholangiocarcinoma (CCA) is an aggressive malignant tumor of the biliary tract that is often challenging to diagnose and treat^1^. It is the second most common primary malignancy, and its incidence has increased significantly in recent decades^2,3^. Although our understanding of the molecular biology of CCA has greatly increased and treatment options for CCA have improved in the last few decades, the prognosis of CCA is poor. Most patients have advanced disease and recurrence after resection^4^. Thus, increasing attention has been focused on traditional Chinese herbal medicines to explore new treatments for patients with CCA. Danshen (*Salvia miltiorrhiza*) is a traditional Chinese medicine. Tanshinone IIA (Tan-IIA) is extracted from Danshen and has been reported to exhibit a number of pharmacological activities against cancer^5,6^. Numerous studies have shown that the anticancer potential of Tan-IIA has also been implicated including hepatocellular carcinoma^7^ and pancreatic cancer^8,9^. However, the specific mechanism of the anti-tumor effect of Tan-IIA on CCA cells remains to be elucidated.

Cyberpharmacology is a relatively new discipline that is based on the theory of systems biology, which allows for a more accurate study of the relevant therapeutic targets of a drug or human disease^10^. In this study, a network pharmacological analysis identified common targets for Tan-IIA and Cholangiocarcinoma disease, and KEGG and GO analyses revealed that these common targets were enriched in pathways associated with PI3K-Akt. It is well known that in human cancers, phosphatidylinositol 3-kinase (PI3K)/protein kinase B (AKT)/mammalian target of rapamycin (mTOR) pathway is the most common aberrant kinase cascade signaling pathway that promotes tumor cell proliferation through the activation of growth factor receptors such as insulin-like growth factor 1 receptor (IGF1R), vascular endothelial growth factor receptor (VEGFR), and epidermal growth factor receptor (EGFR)^11^. Therefore, we selected the PI3K/Akt/mTOR signaling pathway for further study. The present study suggested that Tan-IIA inhibited the proliferation, invasion, and migration of Cholangiocarcinoma cells by inhibiting the PI3K/Akt/mTOR pathway. Furthermore, Tan-IIA upregulated the level of cleaved caspase-3 and suppressed the expression of Bcl2, which ultimately induced apoptosis of Cholangiocarcinoma cells.

## Materials and methods

### Ethical approval

The protocols of all animal experiments were reviewed and approved by the Research Ethics Committee of Qingdao Municipal Hospital (The ethic approval ID: 028), and all animal experimental studies were conducted in accordance with the ARRIVE guidelines. All experiments in the text were carried out in compliance with the relevant rules and regulations and under the supervision and guidance of the Ethics Committee of Qingdao Municipal Hospital.

### Chemicals and reagents

Tan-IIA (Sigma-Aldrich, purity > 97%). The PI3K agonist 740y-p was purchased from MCE (Shanghai, China), Annexin V-FITC/PI staining kit (absin, abs50001, China), Cell Counting Kit-8 (CCK-8) reagent (APExBIO, K1018, USA), Matrigel glue (BD Biosciences, NJ, USA), and BCA protein analysis kit (Beyotime, Shanghai, China), and ECL reagent (Millipore, Massachusetts, USA). Primary antibodies included anti-PI3K (20584-1-AP, Proteintech, China) and anti-Akt antibody (10176-2-AP, Proteintech, China), anti-mTOR (2983, CST, USA), anti-p-Akt (4060, CST, USA), anti-p-mTOR (5536, CST, USA), anti-Bax (2774, CST, USA), anti- Bcl2 (3498, CST, USA), and anti-Caspase-3 (9662, CST, USA) and anti-Cleaved-Caspase-3 (9661, CST, USA).

### Cell lines and culture conditions

Human Cholangiocarcinoma cell lines, HuCCT-1 and RBE, were purchased from the Chinese Academy of Sciences (Shanghai) Cell Bank. The cells were maintained in Dulbecco’s Modified Eagle Medium (DMEM, Hyclone, USA) containing 10% fetal bovine serum (FBS, Excellbio, USA) and 1% penicillin–streptomycin (HyClone, UT, USA). The cells were cultured at 37 °C in an incubator containing 5% CO_2_.

### Cell viability

The cells were incubated overnight in 96 well plates at a density of 5 × 10^3^ cells per well. Then the cells were treated with different concentrations (0, 5, 10, 20, and 30 µg/mL) of Tan-IIA at different times (12, 24, 48, and 72 h), and a 10 µL CCK8 reagent was added (APExBIO, K1018, USA) to each well. After incubation at 37 °C for 1 h, the optical density (OD) was measured at 450 nm. IC50 was calculated using GraphPad Prism 7.0 software.

### Plate cloning

Both cell lines were cultured in a well plate at a density of 300 cells per well. The cells were gently rotated to disperse the cells evenly. After 6 h, the Cholangiocarcinoma cells were treated with Tan-IIA (0, 5, 10, 20, and 30 µg/mL) and incubated in a cell culture incubator at 37 °C with 5% CO_2_ for two weeks. Next, the cells were washed with PBS three times. Subsequently, 4% paraformaldehyde was used to fix the cells for 15 min. The cells were then stained with crystal violet for 10 min. Subsequently, the staining solution was washed off with PBS. The six well plates were then inverted and an overlay of transparency sheet with a grid was performed and the clones was manually counted directly with the naked eye: clone formation rate = (number of clones/number of inoculated cells) × 100%.

### Scratch-induced wound healing assay

Plated in six well culture dishes were 4 × 10^5^ of Cholangiocarcinoma cells. After 24 h, a 200 μL tip was used to wound confluent cells. The detached cells were washed with PBS three times, and the cells were incubated with Tan-IIA (HuCCT-1: 27 µg/mL, RBE: 49 µg/mL) for 24 h. Cell migration images were recorded using an inverted microscope. The results of the scratch experiment were obtained using the formula wound closure rate = post-healing area/initial wound area. All experiments were repeated three times.

### Transwell invasion assay

Cell invasion was detected in a 24-well Transwell chamber. The upper chamber was precoated with 50 µL Matrigel. Cholangiocarcinoma cells (5 × 10^4^) were suspended in 100 µL serum-free medium supplemented with or without Tan-IIA (HuCCT-1: 27 µg/mL, RBE: 49 µg/mL) and were seeded into the upper chamber. At the same time, 600 µL DMEM containing 10% FBS was placed into the lower chamber. After 24 h incubation, Cholangiocarcinoma cells on the upper side of the membrane were wiped with a clean swab. Then, the cells on the underside of the membrane were fixed with methanol and stained with crystalline violet. Nine regions were randomly selected to count the number of invading cells using an inverted microscope.

### Apoptosis determined by the Annexin-V-FITC/PI

Cholangiocarcinoma cells were cultured in six well plates at a density of 2 × 10^5^ cells per well. The cells were then treated with or without Tan-IIA (HuCCT-1: 27 µg/mL, RBE: 49 µg/mL) for 24 h. Then, cells were digested by trypsin without EDTA and harvested and then washed twice with cold PBS. Subsequently, the cells were suspended in binding buffer and stained with 5 μL Annexin V-FITC for 30 min. Next, PI (10 µL) was added for 15 min at room temperature in darkness. Finally, the samples were analyzed by Accuri C6 flow cytometry (BD Biosciences, CA, USA).

### Identification of common targets for Tan-IIA and Cholangiocarcinoma

The targets of Tan-IIA were identified using the TCMSP database (https://tcmspw.com/tcmsp.php), and then gene annotation of the TCM targets was conducted on the Uniprot website (https://www.uniprot.org/). From OMIM (https://omim.org/), GeneCards (https://www.genecards.org/), PharmGKB (https://www.pharmgkb.org/), and TTD (http://db.idrblab.net/ttd/) databases were used to obtain relevant targets of Cholangiocarcinoma, and the VennDiagram package^12^ in R software (version number: 4. 0. 0) was used to analyze the common target genes of both.

### Protein–protein interaction (PPI) network

For building a protein–protein interaction (PPI) network, the common targets of Tan-IIA and Cholangiocarcinoma were entered into the string website (https://string-db.org/) to construct PPI plots, and then Cytoscape software was used to construct network pharmacograms.

### GO and KEGG pathway analysis

Gene Ontology (GO) is an international standard classification system for gene function, consisting of three major components: cellular components, molecular function, and biological processes^13^. To investigate the gene functions involved in common targets, we ran GO functional enrichment using the clusterProfiler package^14^ in R software (version number: 4. 0. 0). The analysis of common target-associated pathways between Tan-IIA and Cholangiocarcinoma was performed using Kyoto Encyclopedia of Genes and Genome (KEGG)^15,16^.

### Western blotting

Cholangiocarcinoma cells were lysed in RIPA for 30 min at 4 °C and total protein was extracted by centrifugation for 20 min. Then, BCA protein analysis reagent was used to determine protein concentrations. Protein extracts were boiled for 5 min. After resolution using SDS/PAGE (10%), proteins were transferred to PVDF membranes (Millipore, Bedford, MA, USA). At room temperature, the membranes were sealed with 5% bovine serum albumin (BSA) for 2 h. Diluted primary antibody: Akt (1: 1000), phospho (p)-Akt (1: 2000), PI3K (1:1000), mTOR (1: 1000), phospho (p)-mTOR (1: 2000) (CST, CA, USA), and β-actin(1:5000). Subsequently, the target membranes were incubated with the specific primary antibodies overnight at 4 °C. Membranes were then washed with TBST three times and then diluted HRP-coupled secondary antibodies were added and incubated for 2 h at room temperature. Finally, immunocomplexes were detected using an ECL detection reagent (Millipore, MA, USA). The measurement dates were obtained from three separate experiments. The intensity of each band was determined using ImageJ software.

### In vivo experiments

NOD-SCID (NOD CB17-Prkdcscid/NcrCrl, male, 5 weeks of age) mice were provided by Beijing Vital River Lab-oratory Animal Technology Co., Ltd (Beijing, China). All mice were placed in a 12-h light/dark cycle at 25 +  −1 °C and 56% humidity with free access to food and water. The initial body weights of these mice ranged from 20 to 23 g. Following subcutaneous injection of 2 × 10^6^ HuCCT-1 Cholangiocarcinoma cells into the back of 15 NOD-SCID mice, the mice were divided into three groups: the control group (n = 5), the Tan-IIA (50 mg/kg) treatment group (n = 5), and the Tan-IIA (50 mg/kg) combined with 740y-p (10 mg/kg) treatment group. Tan-IIA was diluted with DMSO: Methanol: Hydroxypropyl-β-cydodextrin (HP-β-CD) = 1: 1: 1. 740y-p was dissolved in the same way. Seven days after the injection of HuCCT-1 Cholangiocarcinoma cells, drugs were injected intraperitoneally into the experimental groups of mice on every other day, and normal saline was given to the control group. Mice were killed at day 21 of inoculation with tumor cells. All mice were executed by dislocation of the cervical vertebrae. Tumor volumes were measured every 3 days before execution.

### Statistical analysis

The statistical software GraphPad 7 was used for data analysis. The experimental results listed in the article represent the dates of at least three separate replicate experiments. Dates are shown as mean and standard deviation. Student’s t-test was used for differences between two groups. Multiple group comparisons were made using one-way ANOVA. Differences were considered statistically significant at P values of less than 0.05.

## Results

### Effects of Tan-IIA on Cholangiocarcinoma cells proliferation, migration, colony formation, and invasion

**Figure 1:**
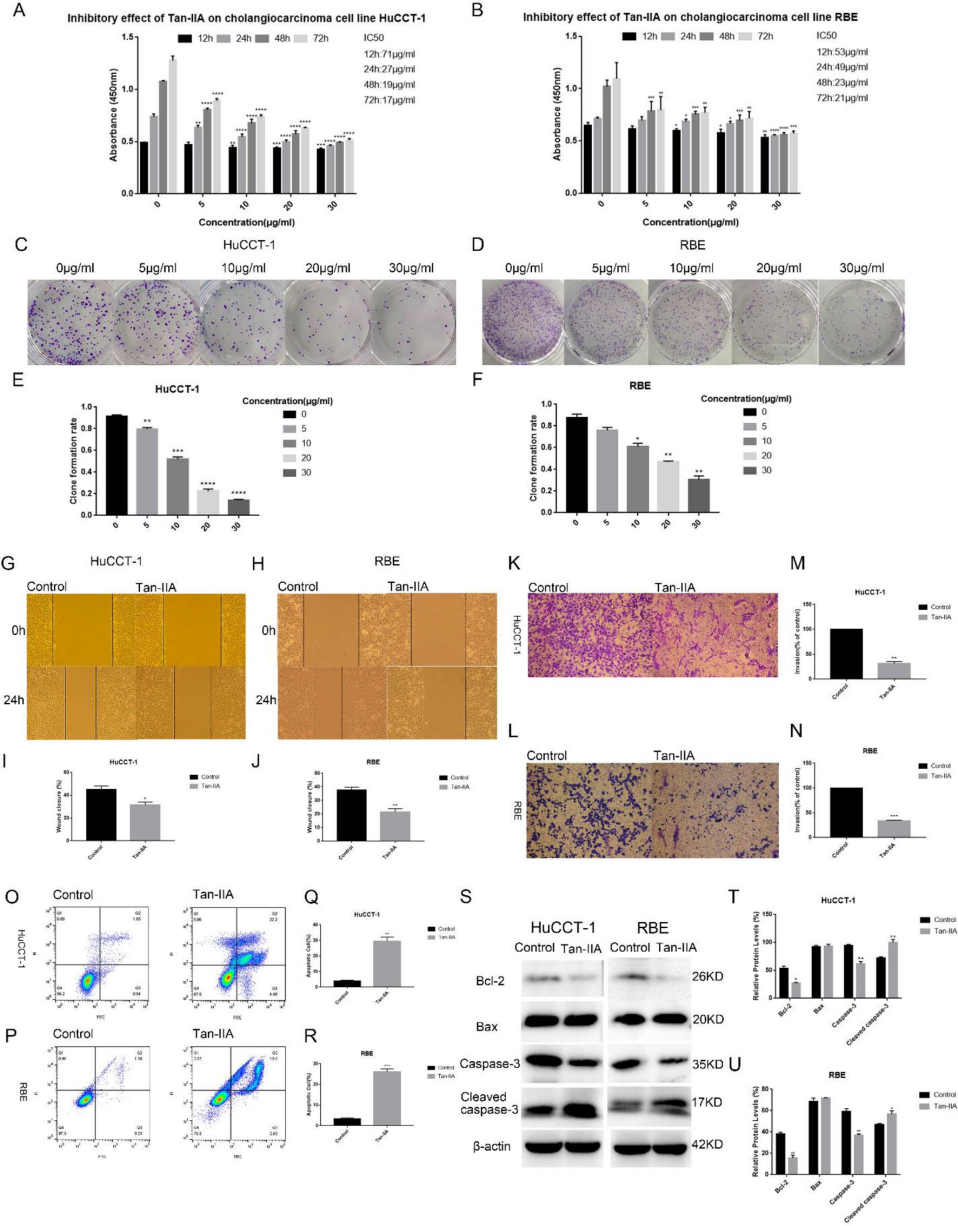
Cytotoxic effect of Tan-IIA on Cholangiocarcinoma cells. (**A**, **B**) Different concentrations (0, 5, 10, 20, and 30 µg/ml) of Tan-IIA were co-cultured with Cholangiocarcinoma cells for 12, 24, 48, and 72 h, and then the cytotoxic effect of Tan-IIA on Cholangiocarcinoma cells were detected by the CCK8 method. Cell viability histogram was made according to the formula (%) = [(experimental group OD value) − (blank group OD value)]/[(control group OD value) − (blank group OD value)] × 100%, and IC50 values were calculated for each time period. (**C**–**F**) Plate clone formation experiments were performed, Cholangiocarcinoma cells were co-cultured with Tan-IIA at concentrations of (0, 5, 10, 20, and 30 µg/mL) for two weeks and the number of clones was counted. Tan-IIA inhibits the migration and invasion of Cholangiocarcinoma cells. (**G**–**J**) Tan-IIA (24 h IC50 concentration) was co-cultured with Cholangiocarcinoma cells, then after scratching the cell layer for 24 h, cell migration by wound healing assay was determined. (**K**–**N**) The effect of Tan-IIA on the invasive ability of Cholangiocarcinoma cells was measured by the Transwell method. After crystalline violet staining, cell images were taken with an inverted microscope. Tan-IIA induced apoptosis of Cholangiocarcinoma cells. (**O**–**R**) The IC50 concentration for 24 h of Tan-IIA was selected and co-cultured with Cholangiocarcinoma cells for 24 h. Annexin V-FITC/PI double staining was used to detect apoptosis. (**S**–**U**) Western blotting to detect the effect of Tan-IIA on the expression of Bax, Bcl-2, as well as caspase-3 and cleaved caspase-3. Compared with control, *p < 0.05; **p < 0.01; ***p < 0.001; ****p < 0.0001.

To explore the effect of Tan-IIA on Cholangiocarcinoma cells, we incubated Cholangiocarcinoma cells at various concentrations (0, 5, 10, 20, and 30 µg/mL) of Tan-IIA for 12, 24, 48, and 72 h. The effect of Tan-IIA on the proliferation of Cholangiocarcinoma cells was then detected by CCK8 (Fig. 1A,B). This indicated that compared with the control group, Tan-IIA inhibited the proliferation of Cholangiocarcinoma cells in a time- and dose-dependent manner (Fig. 1A,B). At the same time, the plate cloning experiment demonstrated that Tan-IIA significantly suppressed Cholangiocarcinoma cells growth compared with the control group (Fig. 1C–F). Scratch wound assay showed that Tan-IIA treatment significantly suppressed the motility of Cholangiocarcinoma cells, as determined by the migration area (Fig. 1G–J). We further assessed the role of Tan-IIA in invasion by a transwell assay. The results showed that Tan-IIA significantly decreased the invasion capacity of Cholangiocarcinoma cells (Fig. 1K–N). In addition, the apoptotic rate detected by flow cytometry analysis showed that Tan-IIA could promote the apoptosis of Cholangiocarcinoma cells (Fig. 1O–R). Next, western blot analysis revealed that compared with the control group, the expression of Bcl-2 was significantly decreased. However, the protein level of BAX was not altered by Tan- IIA. In addition, the results showed that the expression of caspase-3 was markedly decreased, while the cleaved caspase-3 was markedly upregulated induced by Tan-IIA (Fig. 1S–U).

### Common targets of Tan-IIA and Cholangiocarcinoma effects

**Figure 2:**
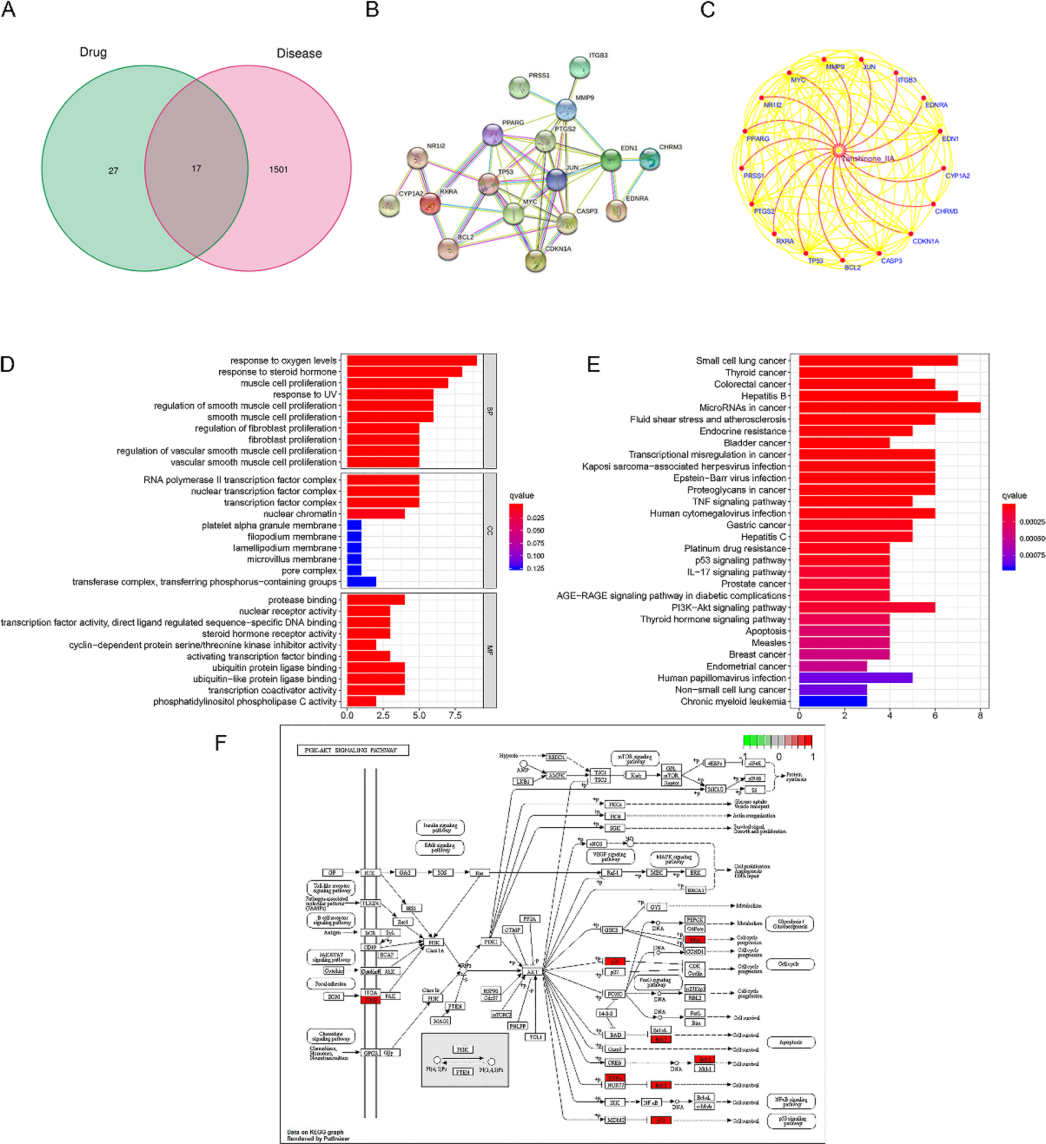
Identifying common targets for Tan-IIA and Cholangiocarcinoma. (**A**) Venn diagrams were created by using the VennDiagram package^12^ in R software (version number: 4. 0. 0), depicting the intersection of Tan-IIA and Cholangiocarcinoma targets. (**B**) PPI network was constructed using 17 common targets of Cholangiocarcinoma and Tan-IIA. (**C**) Cytoscape plotted the network pharmacology, using red and yellow lines to indicate the interactions between Tan-IIA and targets and target-to-target interactions. GO and KEGG analysis of common targets. (**D**) GO functional enrichment was ran by using the clusterProfiler package^14^ in R software (version number: 4. 0. 0) and GO analysis of the enriched biological functions of the common target genes was performed (counts ≥ 30). (**E**) KEGG^15,16^ analysis of signaling pathways enriched by common target genes (counts ≥ 30). (**F**) Map of PI3K/AKT pathway obtained from KEGG analysis.

Relevant targets for Cholangiocarcinoma were obtained from OMIM, GeneCards, PharmGKB, and TTD databases, and the target of Tan-IIA was found from the TCMSP database. After cross-analysis, 17 common drug-disease-related targets were identified (Fig. 2A). The screened targets were then used to construct the PPI network through the string website (Fig. 2B). Using the data tables obtained from the PPI network, the network pharmacology map was entered into Cytoscape software (Fig. 2C). The relationship between the common target genes and the relationship between the genes and Tan-IIA can be clearly seen in the figure. Then, we analyzed the GO and KEGG results of the common targets of Tan-IIA and Cholangiocarcinoma. Based on the 17 common targets of Tan-IIA and Cholangiocarcinoma, we used R software to analyze the top ten terms of the three major categories of BP, CC, and MF enriched by GO (Fig. 2D), and the results of GO analysis showed that the common targets of Tan- IIA and Cholangiocarcinoma were closely related to cell proliferation. Among the top 30 terms in KEGG analysis (Fig. 2E), we found that the PI3K-AKT pathway was more significantly associated with proliferation (Fig. 2F).

### Tan-IIA inhibited activation of PI3K/AKT/mTOR

**Figure 3:**
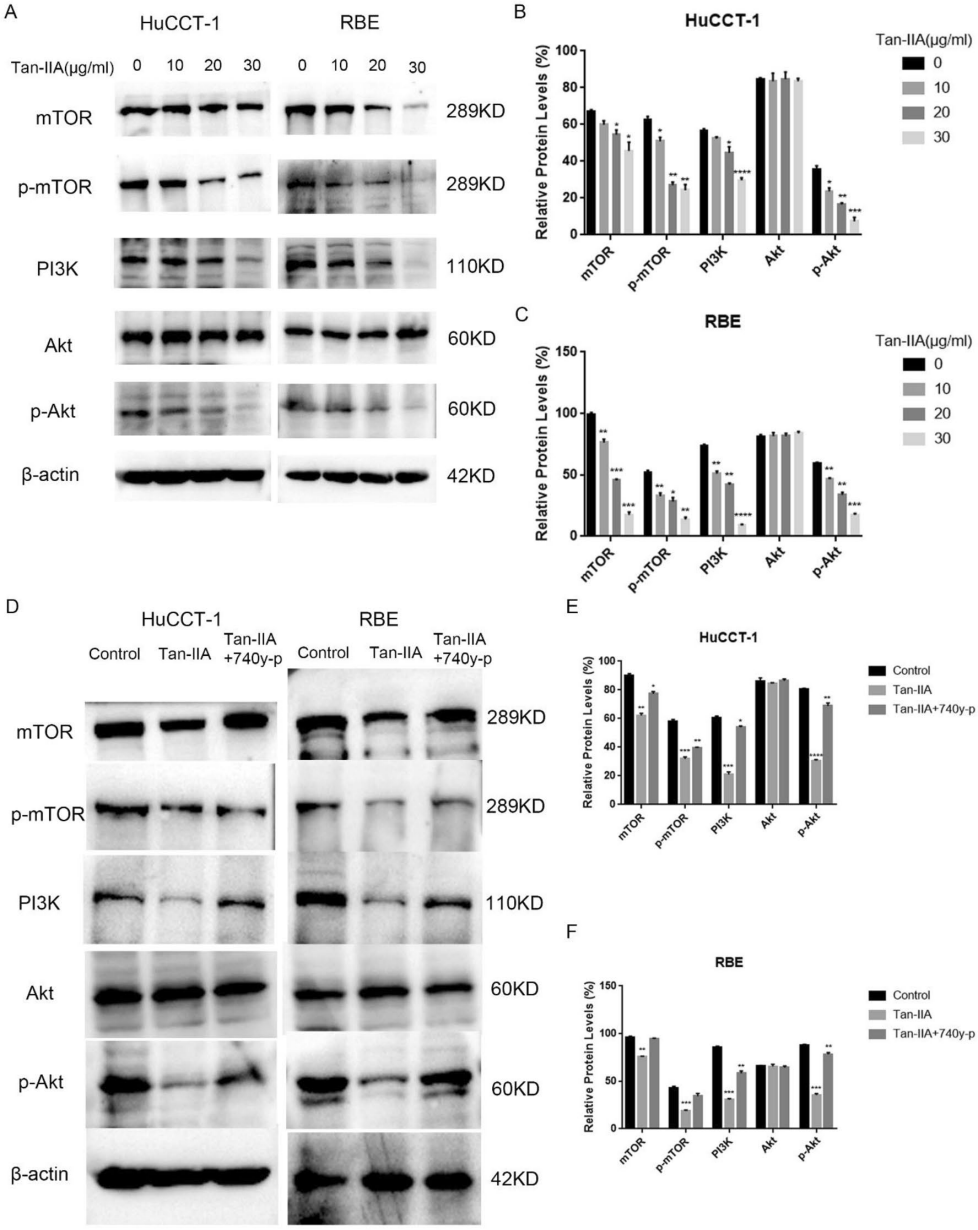
Tan-IIA can inhibit the PI3K-Akt-mTOR pathway. (**A**–**C**) Tan-IIA at concentrations of 0, 10, 20, and 30 µg/mL was added to fresh medium and co-cultured with Cholangiocarcinoma cells for 24 h. Next, the protein expression levels of PI3K, Akt, mTOR, p-Akt, and p-mTOR were measured using western blots. (**D**–**F**) After the addition of 740y-p (10 µg/mL) to the system in which Tan-IIA (24 h IC50 concentration) and Cholangiocarcinoma cells were co-cultured. The inhibitory effect of Tan-IIA on PI3K-Akt-mTOR pathway was diminished under the influence of PI3K agonists. Compared with control, *p < 0.05; **p < 0.01; ***p < 0.001; ****p < 0.0001.

Western blotting was used to detect the expression of PI3K, Akt, p-Akt, mTOR, and p-mTOR. The results indicated that Tan-IIA inhibited the expression of PI3K, p-Akt, p-mTOR, and mTOR compared to control group in a dose-dependent manner (Fig. 3A–C). Furthermore, pretreatment of Cholangiocarcinoma cells with the 740 y-p (PI3K agonist) abolished the effects of Tan-IIA on the restrain PI3K, p-Akt, mTOR, and p-mTOR in Cholangiocarcinoma cells (Fig. 3D–F).

### PI3K agonists abolished the effects of Tan-IIA

**Figure 4:**
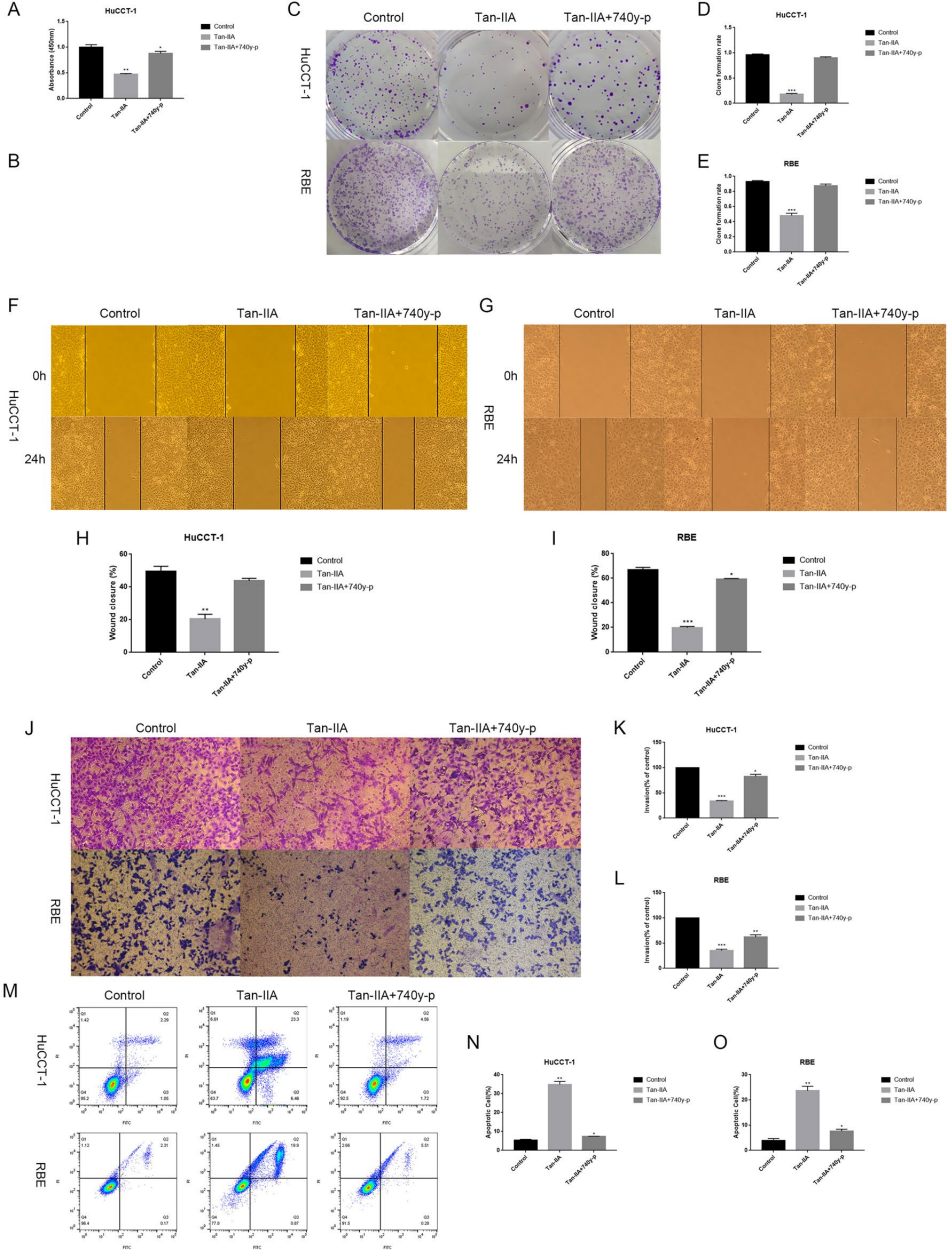
PI3K agonists attenuate the tumor suppressive effect of Tan-IIA. After the addition of 740y-p (10 µg /mL) to the system in which Tan-IIA (24 h IC50 concentration) and Cholangiocarcinoma cells were co-cultured, the tumor suppressive effect of Tan-IIA was attenuated. (**A**–**E**) The inhibitory effect of Tan-IIA on the proliferation of Cholangiocarcinoma cells decreased after the addition of 740y-p. (**F**–**L**) The ability of Tan-IIA to inhibit the invasion and migration of Cholangiocarcinoma cells was reduced after the addition of 740y-p. (**M**–**O**) The apoptosis-inducing effect of Tan-IIA on Cholangiocarcinoma cells was diminished after 740y-p was added. Compared with control, *p < 0.05; **p < 0.01; ***p < 0.001.

To further demonstrate that Tan-IIA affects the growth of Cholangiocarcinoma cells by suppressing the PI3K/Akt/mTOR signaling pathway, Cholangiocarcinoma cells were pretreated with 740 y-p (PI3K agonist) or without it in the presence of Tan-IIA. These results confirm that PI3K agonists could attenuate the tumor suppressive effect of Tan-IIA. The data showed that the effect of Tan-IIA inhibited cell proliferation, migration, and invasiveness, and promoted apoptosis was reversed by 740 y-p treatment (Fig. 4A–O).

### Tan-IIA suppresses tumor growth in vivo

**Figure 5:**
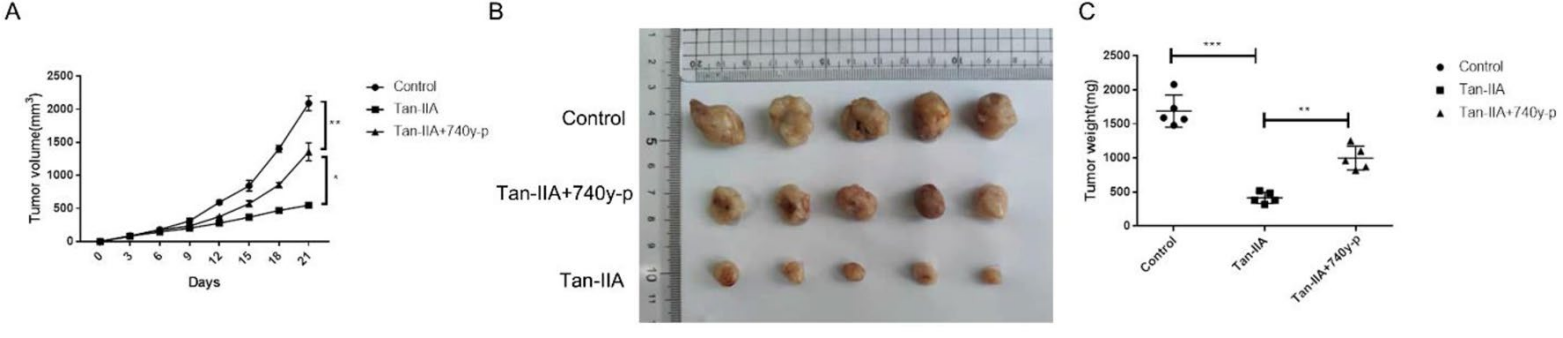
Tan-IIA suppresses tumor growth in vivo. Mice were treated with normal saline, Tan-IIA (50 mg/kg) and Tan-IIA (50 mg/kg) combined with 740y-p (10 mg/kg) for 3 weeks, tumor volume was measured every 3 days (n = 5). (**A**) After treatment with normal saline, Tan-IIA and Tan-IIA combined with 740y-p for 3 weeks, tumors in these groups were removed, tumor pictures were captured (**B**) and tumors were weighed (**C**). Compared with control, *p < 0.05; **p < 0.01; ***p < 0.001.

We used HuCCT-1 cell xenograft model to investigate the anti-tumor effect of Tan-IIA and the role of PI3K/Akt/mTOR signaling pathway in the anti-tumor effect of Tan-IIA. After 3 weeks of treatment with 50 mg/kg Tan-IIA or 50 mg/kg Tan-IIA combined with 10 mg/kg 740y-p. The results showed that Tan-IIA also had a significant anti-tumor effect in vivo. However, the anti-tumor effect of Tan-IIA was largely attenuated by 740y-p (Fig. 5A–C).

## Discussion

Tan-IIA was one of the main components of Danshen. Accumulated evidence from preclinical and clinical studies has confirmed that Tan-IIA has good anti-tumor properties^17^. Several studies have shown that it can inhibit the growth of various tumor cell lines, including liver cancer, pancreatic cancer, and colorectal cancer^7,8,18^. Nevertheless, the inhibitory effect of Tan-IIA on CCA cells and its underlying mechanisms are unknown. Consistent with the previous findings, this study showed that Tan-IIA could inhibit malignant growth, migration, and invasion and promote apoptosis of Cholangiocarcinoma cells, providing a new understanding of the role of Tan-IIA in the treatment of CCA.

To investigate the mechanism by which Tan-IIA inhibits Cholangiocarcinoma, we analyzed the possible pathways that Tan-IIA inhibited the malignant proliferation of Cholangiocarcinoma cells through network pharmacology. KEGG analysis showed that Tan-IIA affected the expression of the PI3K/Akt pathway in Cholangiocarcinoma cells. Several studies have shown that elevated expression of PI3K- associated proteins is considered a hallmark of cancer^19^. The PI3K/Akt pathway is closely related to cancer progression in many types of human cancers, including lung cancer, stomach cancer, liver cancer, and pancreatic cancer. Previous studies have shown that the PI3K/Akt pathway plays a significant role in inhibiting tumor proliferation, invasion, migration, and apoptosis^20^. It has been reported that the PI3K/Akt signaling pathway is extremely important in CCA development and progression^21^. In the present study, we detected the expression of PI3K/Akt proteins in Cholangiocarcinoma cells. Consistent with KEGG analysis, we observed that Tan-IIA significantly inhibited the levels of PI3K and p-Akt compared with those in the control group in Cholangiocarcinoma cells.

Recent studies have shown that the activation of the PI3K/Akt pathway can activate or inhibit a variety of downstream target proteins, such as mTOR, Bad, Caspase9 and GSK-3. And the PI3K/Akt/mTOR signaling cascade has a major impact on cell proliferation and survival as well as cell cycle regulation^22,23^. In this pathway, PI3K is activated and further activates Akt proteins located on the plasma membrane, which eventually phosphorylate Akt, and then p-Akt activates various regulators downstream of the pathway, including mTOR^24^. mTOR is the main downstream effector of the PI3K/Akt pathway. Activated mTOR is associated with cell proliferation and survival^25^. Phosphorylation of ribosomal protein S6 kinase (S6K) and eukaryotic translation initiation factor 4E binding protein 1 (4E-BP1) can be mediated by activated mTORC1, which leads to the re-release of eukaryotic translation initiation factor 4E (EIF4E). These factors directly lead to protein translation and cell cycle progression. In addition, mTORC2 can act as an activator of Akt, forming a positive feedback loop between Akt and mTOR, further promoting cell proliferation and survival^26^. It is well documented that Tan-IIA plays an anti-tumor role by regulating the PI3K/Akt/mTOR signaling pathway. Tan-IIA can block the Ras/Raf/MEK/ERK and PI3K/Akt/mTOR pathways, thereby inhibiting the malignant growth of human pancreatic cancer cells^8^. Tan-IIA has also been reported to induce apoptosis and autophagy in acute monocytic leukemia by inhibiting the PI3K/Akt/mTOR signaling pathway^27^. In the present study, the results revealed that Tan-IIA significantly inhibited the level of PI3K, p-Akt, mTOR and p-mTOR protein in Cholangiocarcinoma cells. Following the addition of the PI3K agonist (740 y-p), the levels of p-Akt, mTOR and p-mTOR increased, and 740 y-p eliminated the inhibitory effect of Tan-IIA on Cholangiocarcinoma cells. These results indicated that Tan-IIA inhibited the proliferation, migration, and invasion of Cholangiocarcinoma cells by inhibiting the PI3K/AKT/mTOR pathway.

Apoptosis is an important manifestation of cell death, and promoting tumor cell apoptosis plays a crucial role in inhibiting the development and progression of tumors^28^. Previous studies have shown that the PI3K/Akt/mTOR signaling pathway plays an essential role in regulating cell apoptosis^29^. The novel finding in this study is that inhibition of PI3K by 740 y-p eliminated the effect of Tan-IIA on the proapoptosis of Cholangiocarcinoma cells. Our findings indicated that Tan-IIA induced Cholangiocarcinoma cells apoptosis via inhibition of the Akt/mTOR pathway. In addition, the Bcl-2 family and the caspase family have been reported to participate in the mitochondria-mediated pathway of apoptosis. Caspase-3 belongs to the cysteine-aspartic acid protease (caspase) family, which regulates apoptosis by interacting with caspase-8 and caspase-9^30^. Whereas Bax and Bcl-2 are members of the Bcl-2 gene family, Bcl-2 mainly plays a role in inhibiting apoptosis and can maintain cell survival^31^. Bax, on the other hand, is a pro-apoptotic factor in the Bcl-2 family that can affect the mitochondrial membrane, leading to the release of cytochrome C and the production of reactive oxygen species, in addition, Bax can form heterodimer with Bcl-2 and activate endogenous apoptosis. Therefore, Bax is a major regulator involved in the process of apoptosis^32^. Moreover, compared with the expression of Bax and Bcl-2, respectively, the ratio of Bax/Bcl-2 is more important for apoptosis. In this study, western blotting and flow cytometry assays revealed that Tan-IIA treatment promoted apoptosis and upregulated the level of cleaved caspase-3 and the ratio of Bax/Bcl-2. The results demonstrated that caspase3 and Bax/Bcl2 play critical regulatory roles in the progression of Tan-IIA promoted apoptosis in Cholangiocarcinoma cells.

## Conclusion

In conclusion, our study revealed the potential anti-tumor effects of Tan-IIA in Cholangiocarcinoma cells. Further mechanism studies demonstrated that Tan-IIA promoted apoptosis and suppressed malignant growth, invasion, and migration of Cholangiocarcinoma cells through inhibited PI3K/Akt/mTOR signaling pathway. Furthermore, we suggested that Tan-IIA could be a potent agent for the treatment of CCA.

## Supplementary Information

Supplementary Information.

## References

[1] El-Diwany, R., Pawlik, T. M. & Ejaz, A. Intrahepatic Cholangiocarcinoma. *Surg. Oncol. Clin. N Am.* **28**, 587–599. 10.1016/j.soc.2019.06.002 (2019). 31472907 10.1016/j.soc.2019.06.002

[2] Rizvi, S., Khan, S. A., Hallemeier, C. L., Kelley, R. K. & Gores, G. J. Cholangiocarcinoma—Evolving concepts and therapeutic strategies. *Nat. Rev. Clin. Oncol.* **15**, 95–111. 10.1038/nrclinonc.2017.157 (2018). 28994423 10.1038/nrclinonc.2017.157 PMC5819599

[3] Patel, N. & Benipal, B. Incidence of Cholangiocarcinoma in the USA from 2001 to 2015: A US cancer statistics analysis of 50 states. *Cureus* **11**, e3962. 10.7759/cureus.3962 (2019). 30956914 10.7759/cureus.3962 PMC6436669

[4] Weigt, J. & Malfertheiner, P. Cisplatin plus gemcitabine versus gemcitabine for biliary tract cancer. *Expert Rev. Gastroenterol. Hepatol.* **4**, 395–397. 10.1586/egh.10.45 (2010). 20678012 10.1586/egh.10.45

[5] Che, A. J. *et al.* Separation and determination of active components in Radix *Salviae miltiorrhizae* and its medicinal preparations by nonaqueous capillary electrophoresis. *J. Sep. Sci.* **27**, 569–575. 10.1002/jssc.200301710 (2004). 15335042 10.1002/jssc.200301710

[6] Zhou, L., Zuo, Z. & Chow, M. S. Danshen: An overview of its chemistry, pharmacology, pharmacokinetics, and clinical use. *J. Clin. Pharmacol.* **45**, 1345–1359. 10.1177/0091270005282630 (2005). 16291709 10.1177/0091270005282630

[7] Ma, L. *et al.* Tanshinone IIA mediates SMAD7-YAP interaction to inhibit liver cancer growth by inactivating the transforming growth factor beta signaling pathway. *Aging (Albany NY)* **11**, 9719–9737. 10.18632/aging.102420 (2019). 31711043 10.18632/aging.102420 PMC6874425

[8] Su, C. C. Tanshinone IIA can inhibit MiaPaCa2 human pancreatic cancer cells by dual blockade of the Ras/Raf/MEK/ERK and PI3K/AKT/mTOR pathways. *Oncol. Rep.* **40**, 3102–3111. 10.3892/or.2018.6670 (2018). 30226540 10.3892/or.2018.6670

[9] Huang, C. Y. *et al.* Tanshinone IIA inhibits the growth of pancreatic cancer BxPC3 cells by decreasing protein expression of TCTP, MCL1 and BclxL. *Mol. Med. Rep.* **7**, 1045–1049. 10.3892/mmr.2013.1290 (2013). 23358553 10.3892/mmr.2013.1290

[10] Yuan, H. *et al.* How can synergism of traditional medicines benefit from network pharmacology?. *Molecules*10.3390/molecules22071135 (2017). 28686181 10.3390/molecules22071135 PMC6152294

[11] Liu, P., Cheng, H., Roberts, T. M. & Zhao, J. J. Targeting the phosphoinositide 3-kinase pathway in cancer. *Nat. Rev. Drug Discov.* **8**, 627–644. 10.1038/nrd2926 (2009). 19644473 10.1038/nrd2926 PMC3142564

[12] Chen, H. & Boutros, P. C. VennDiagram: A package for the generation of highly-customizable Venn and Euler diagrams in R. *BMC Bioinform.* **12**, 35. 10.1186/1471-2105-12-35 (2011). 10.1186/1471-2105-12-35 PMC3041657 21269502

[13] Ashburner, M. *et al.* Gene ontology: Tool for the unification of biology. The gene ontology consortium. *Nat. Genet.* **25**, 25–29. 10.1038/75556 (2000). 10802651 10.1038/75556 PMC3037419

[14] Yu, G., Wang, L. G., Han, Y. & He, Q. Y. clusterProfiler: An R package for comparing biological themes among gene clusters. *OMICS* **16**, 284–287. 10.1089/omi.2011.0118 (2012). 22455463 10.1089/omi.2011.0118 PMC3339379

[15] Kanehisa, M. & Goto, S. KEGG: Kyoto encyclopedia of genes and genomes. *Nucleic Acids Res* **28**, 27–30. 10.1093/nar/28.1.27 (2000). 10592173 10.1093/nar/28.1.27 PMC102409

[16] Kanehisa, M., Goto, S., Furumichi, M., Tanabe, M. & Hirakawa, M. KEGG for representation and analysis of molecular networks involving diseases and drugs. *Nucleic Acids Res.* **38**, D355-360. 10.1093/nar/gkp896 (2010). 19880382 10.1093/nar/gkp896 PMC2808910

[17] Ansari, M. A. *et al.* Prospective therapeutic potential of Tanshinone IIA: An updated overview. *Pharmacol. Res.*10.1016/j.phrs.2020.105364 (2020). 33285229 10.1016/j.phrs.2020.105364

[18] Qian, J. *et al.* Tanshinone IIA promotes IL2-mediated SW480 colorectal cancer cell apoptosis by triggering INF2-related mitochondrial fission and activating the Mst1-Hippo pathway. *Biomed. Pharmacother.* **108**, 1658–1669. 10.1016/j.biopha.2018.09.170 (2018). 30372868 10.1016/j.biopha.2018.09.170

[19] Fruman, D. A. *et al.* The PI3K pathway in human disease. *Cell* **170**, 605–635. 10.1016/j.cell.2017.07.029 (2017). 28802037 10.1016/j.cell.2017.07.029 PMC5726441

[20] Zhang, J., Yu, X. H., Yan, Y. G., Wang, C. & Wang, W. J. PI3K/Akt signaling in osteosarcoma. *Clin. Chim. Acta* **444**, 182–192. 10.1016/j.cca.2014.12.041 (2015). 25704303 10.1016/j.cca.2014.12.041

[21] Corti, F. *et al.* Targeting the PI3K/AKT/mTOR pathway in biliary tract cancers: A review of current evidences and future perspectives. *Cancer Treat. Rev.* **72**, 45–55. 10.1016/j.ctrv.2018.11.001 (2019). 30476750 10.1016/j.ctrv.2018.11.001

[22] Polo, M. L. *et al.* Activation of PI3K/Akt/mTOR signaling in the tumor stroma drives endocrine therapy-dependent breast tumor regression. *Oncotarget* **6**, 22081–22097. 10.18632/oncotarget.4203 (2015). 26098779 10.18632/oncotarget.4203 PMC4673148

[23] Xia, P. & Xu, X. Y. PI3K/Akt/mTOR signaling pathway in cancer stem cells: From basic research to clinical application. *Am. J. Cancer Res.* **5**, 1602–1609 (2015). 26175931 PMC4497429

[24] Ersahin, T., Tuncbag, N. & Cetin-Atalay, R. The PI3K/AKT/mTOR interactive pathway. *Mol. Biosyst.* **11**, 1946–1954. 10.1039/c5mb00101c (2015). 25924008 10.1039/c5mb00101c

[25] Tian, T., Li, X. & Zhang, J. mTOR signaling in cancer and mTOR inhibitors in solid tumor targeting therapy. *Int. J. Mol. Sci.*10.3390/ijms20030755 (2019). 30754640 10.3390/ijms20030755 PMC6387042

[26] Chang, W. *et al.* A critical role for the mTORC2 pathway in lung fibrosis. *PLoS ONE* **9**, e106155. 10.1371/journal.pone.0106155 (2014). 25162417 10.1371/journal.pone.0106155 PMC4146613

[27] Zhang, Y. *et al.* Tanshinone IIA induces apoptosis and autophagy in acute monocytic leukemia via downregulation of PI3K/Akt pathway. *Am. J. Transl. Res.* **11**, 2995–3006 (2019). 31217869 PMC6556636

[28] Bold, R. J., Termuhlen, P. M. & McConkey, D. J. Apoptosis, cancer and cancer therapy. *Surg. Oncol.* **6**, 133–142. 10.1016/s0960-7404(97)00015-7 (1997). 9576629 10.1016/s0960-7404(97)00015-7

[29] Manfredi, G. I. *et al.* PI3K/Akt/mTOR signaling in medullary thyroid cancer: A promising molecular target for cancer therapy. *Endocrine* **48**, 363–370. 10.1007/s12020-014-0380-1 (2015). 25115638 10.1007/s12020-014-0380-1

[30] Alnemri, E. S. *et al.* Human ICE/CED-3 protease nomenclature. *Cell* **87**, 171. 10.1016/s0092-8674(00)81334-3 (1996). 8861900 10.1016/s0092-8674(00)81334-3

[31] Hardwick, J. M. & Soane, L. Multiple functions of BCL-2 family proteins. *Cold Spring Harb. Perspect. Biol.*10.1101/cshperspect.a008722 (2013). 23378584 10.1101/cshperspect.a008722 PMC3552500

[32] Gross, A., Jockel, J., Wei, M. C. & Korsmeyer, S. J. Enforced dimerization of BAX results in its translocation, mitochondrial dysfunction and apoptosis. *EMBO J.* **17**, 3878–3885. 10.1093/emboj/17.14.3878 (1998). 9670005 10.1093/emboj/17.14.3878 PMC1170723

