# Author Correction: Tanshinone IIA affects the malignant growth of Cholangiocarcinoma cells by inhibiting the PI3K-Akt-mTOR pathway

**DOI:** 10.1038/s41598-025-99378-x

**Published:** 2025-05-27

**Authors:** Huayuan Liu, Caiyun Liu, Mengya Wang, Dongxu Sun, Pengcheng Zhu, Ping Zhang, Xueying Tan, Guangjun Shi

**Affiliations:** 1 Department of Medicine, Qingdao University, Qingdao, China; 2 Department of Hepatobiliary Surgery, The Affiliated Qingdao Municipal Hospital of Qingdao University, Qingdao, China; 3 Department of Physiology, School of Basic Medicine, Qingdao University, Qingdao, China; 4 Graduate School of Dalian Medical University, Dalian, China; 5 Department of Gynecology, The Affiliated Qingdao Municipal Hospital of Qingdao University, Qingdao, China

Correction to: *Scientific Reports* 10.1038/s41598-021-98948-z, published online 29 September 2021

The original version of this Article contained errors in Figure 4, panels F and G, where the 0h image of the “Tan-IIA+740y-p” group was incorrectly used as the image of the “Tan-IIA group”. The original Fig. 4 and accompanying legend appear below.

**Fig. 4:**
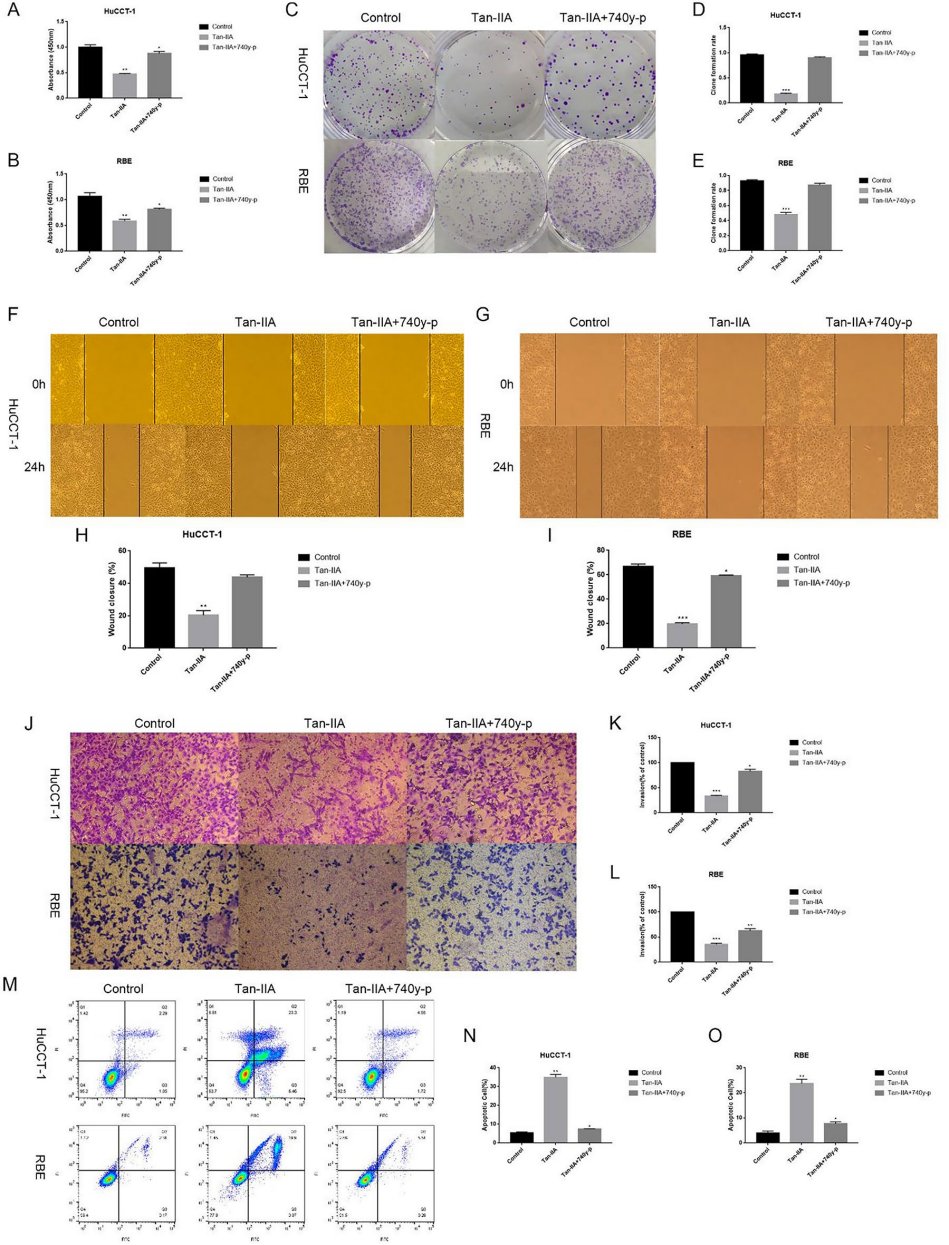
PI3K agonists attenuate the tumor suppressive effect of Tan-IIA. After the addition of 740y-p (10 µg/mL) to the system in which Tan-IIA (24 h IC50 concentration) and Cholangiocarcinoma cells were co-cultured, the tumor suppressive effect of Tan-IIA was attenuated. (**A**–**E**) The inhibitory effect of Tan-IIA on the proliferation of Cholangiocarcinoma cells decreased after the addition of 740y-p. (**F**–**L**) The ability of Tan-IIA to inhibit the invasion and migration of Cholangiocarcinoma cells was reduced after the addition of 740y-p. (**M**–**O**) The apoptosis-inducing effect of Tan-IIA on Cholangiocarcinoma cells was diminished after 740y-p was added. Compared with control, *p < 0.05; **p < 0.01; ***p < 0.001.

The original Article has been corrected.

