# Supplementary material for: Tanshinone IIA affects the malignant growth of Cholangiocarcinoma cells by inhibiting the PI3K-Akt-mTOR pathway
Source: Sci Rep. 2021 Sep 29;11:19268. doi: 10.1038/s41598-021-98948-z (PMC8481305; doi:10.1038/s41598-021-98948-z)
Supplement: Supplementary file 1 — Supplementary Information. [file 41598_2021_98948_MOESM1_ESM.docx]

The first experiment

HuCCT-1

ACTIN


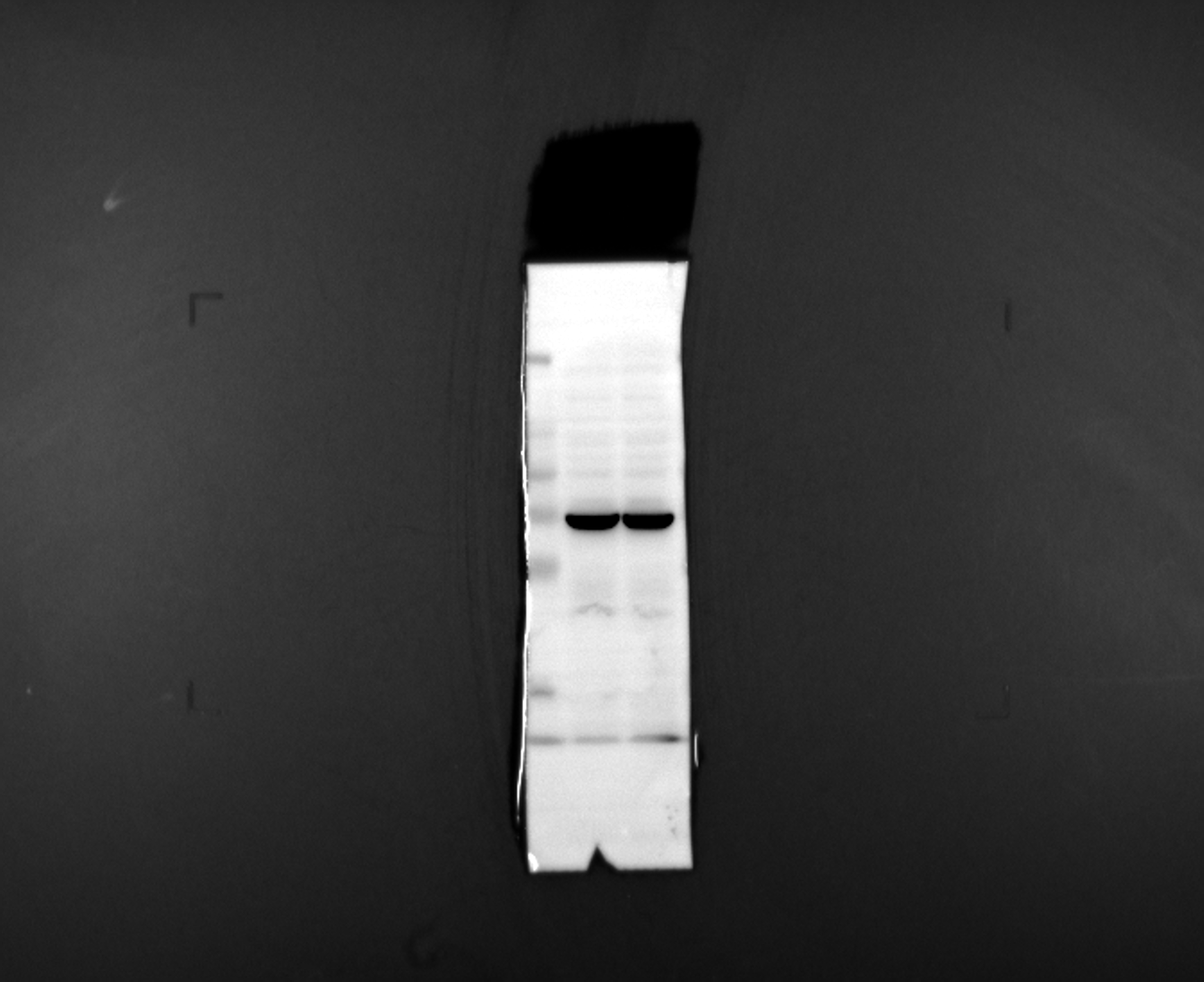

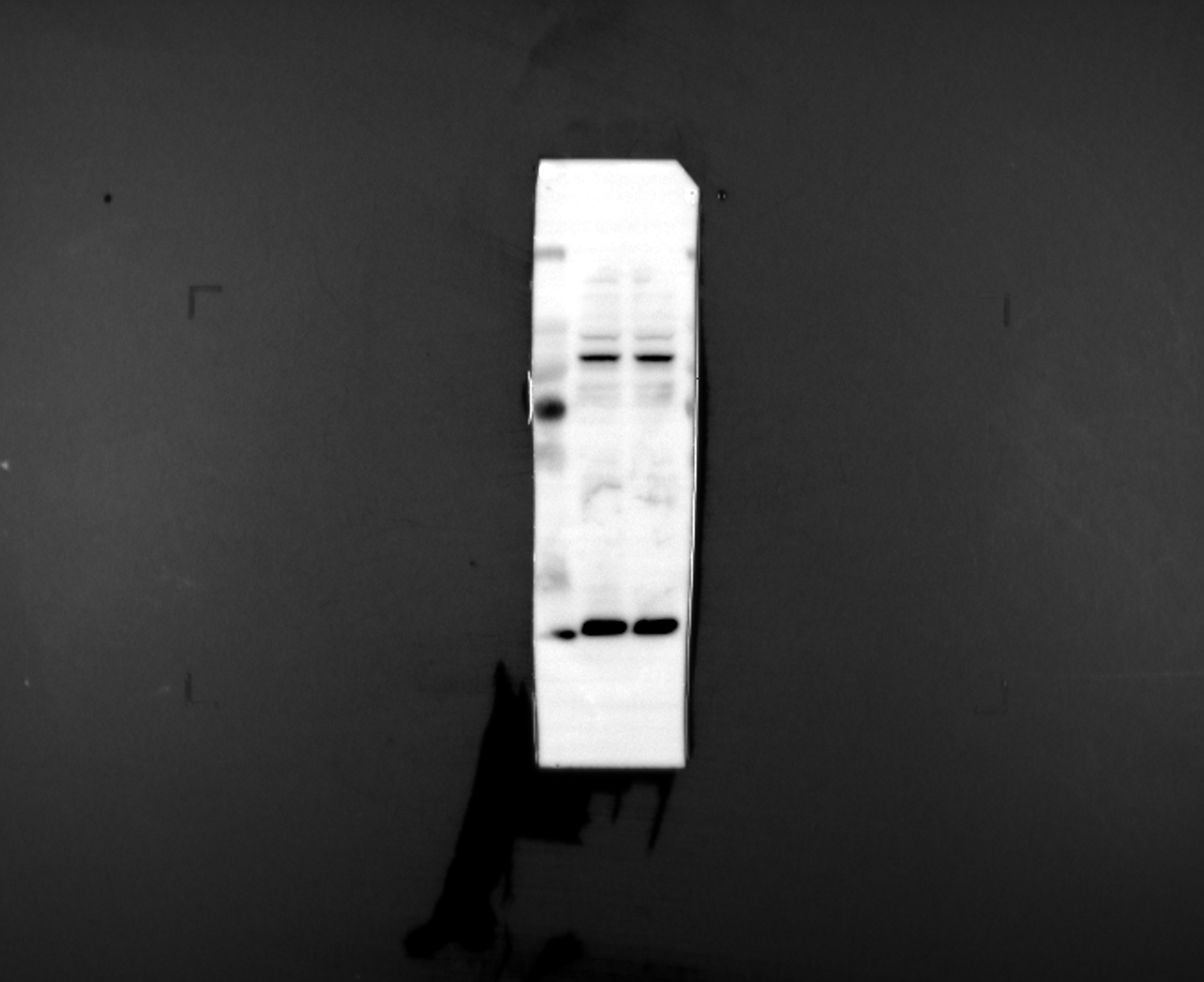


120KDa

70KDa

55KDa

45KDa

35KDa

25KDa

15KDa

Bax

Bcl-2


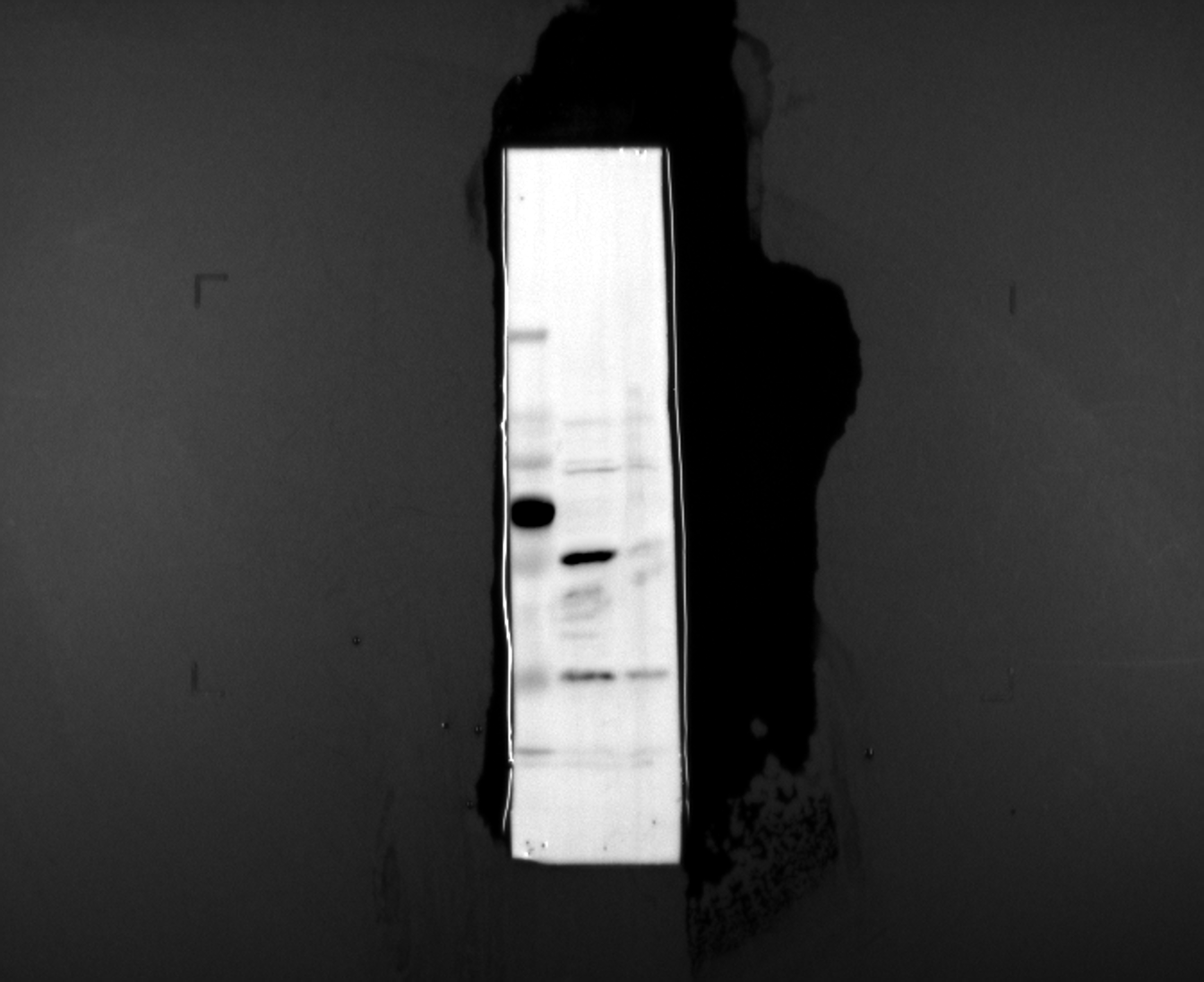


Caspase3


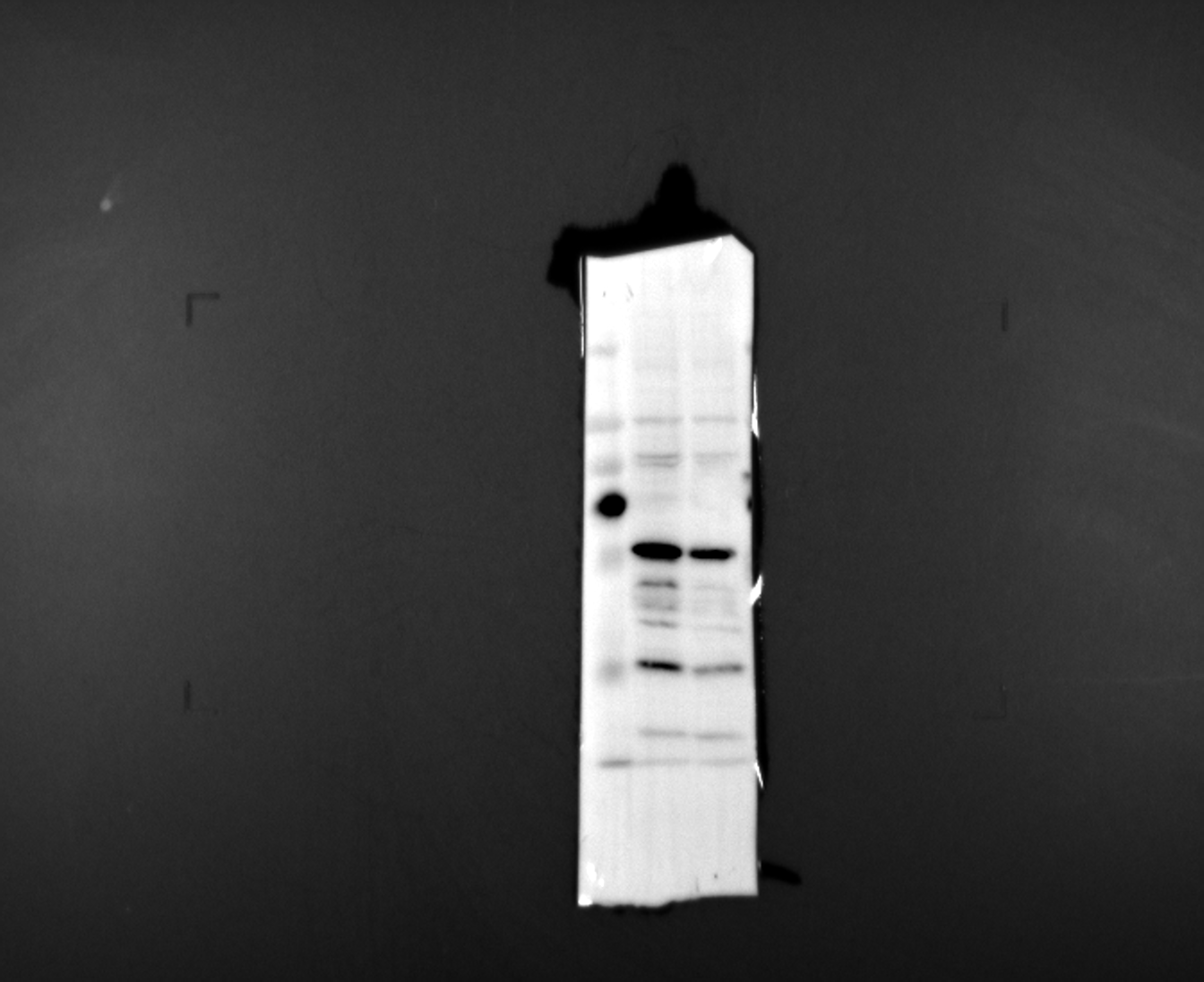


Cleved-caspase3


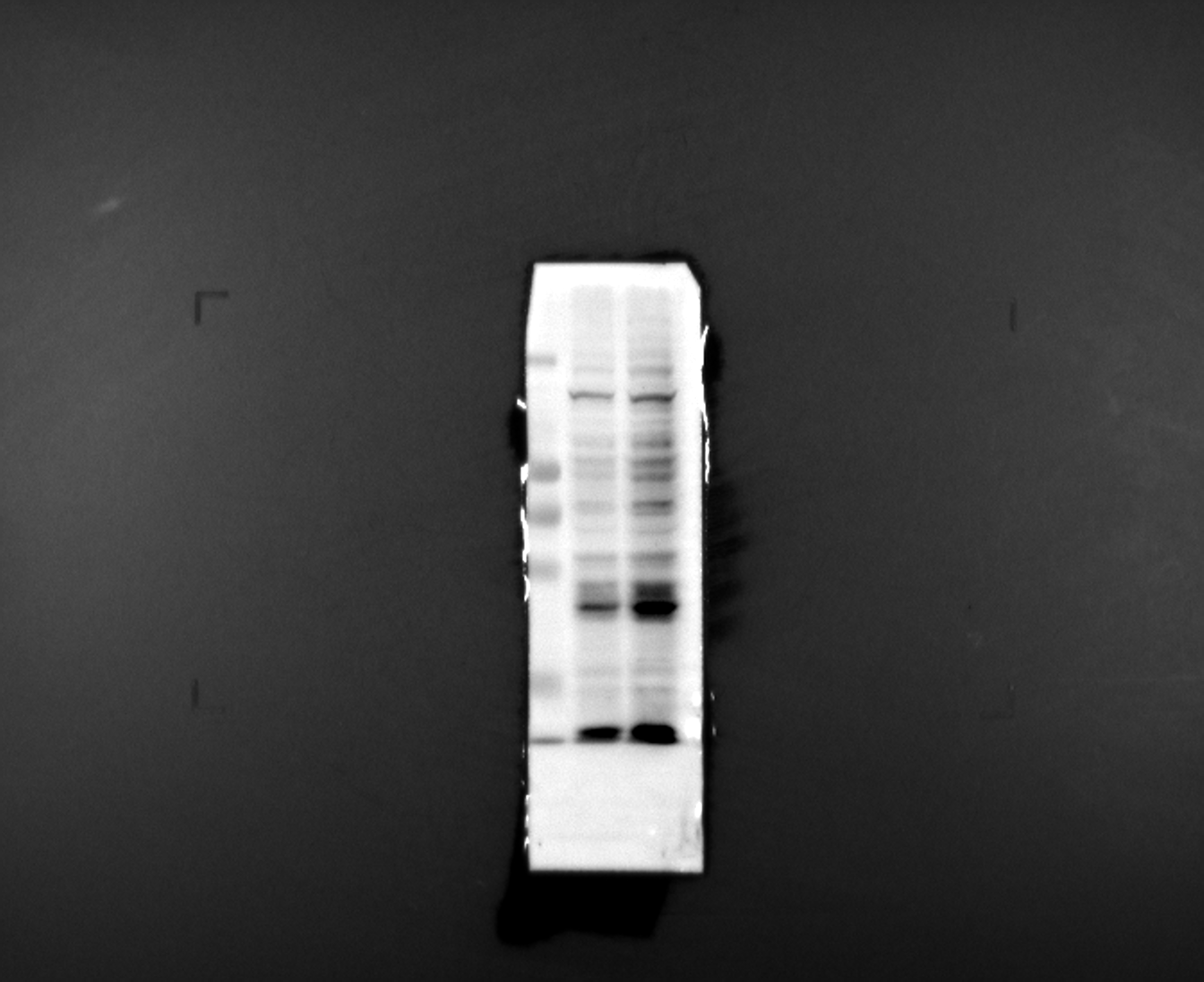


RBE

ACTIN


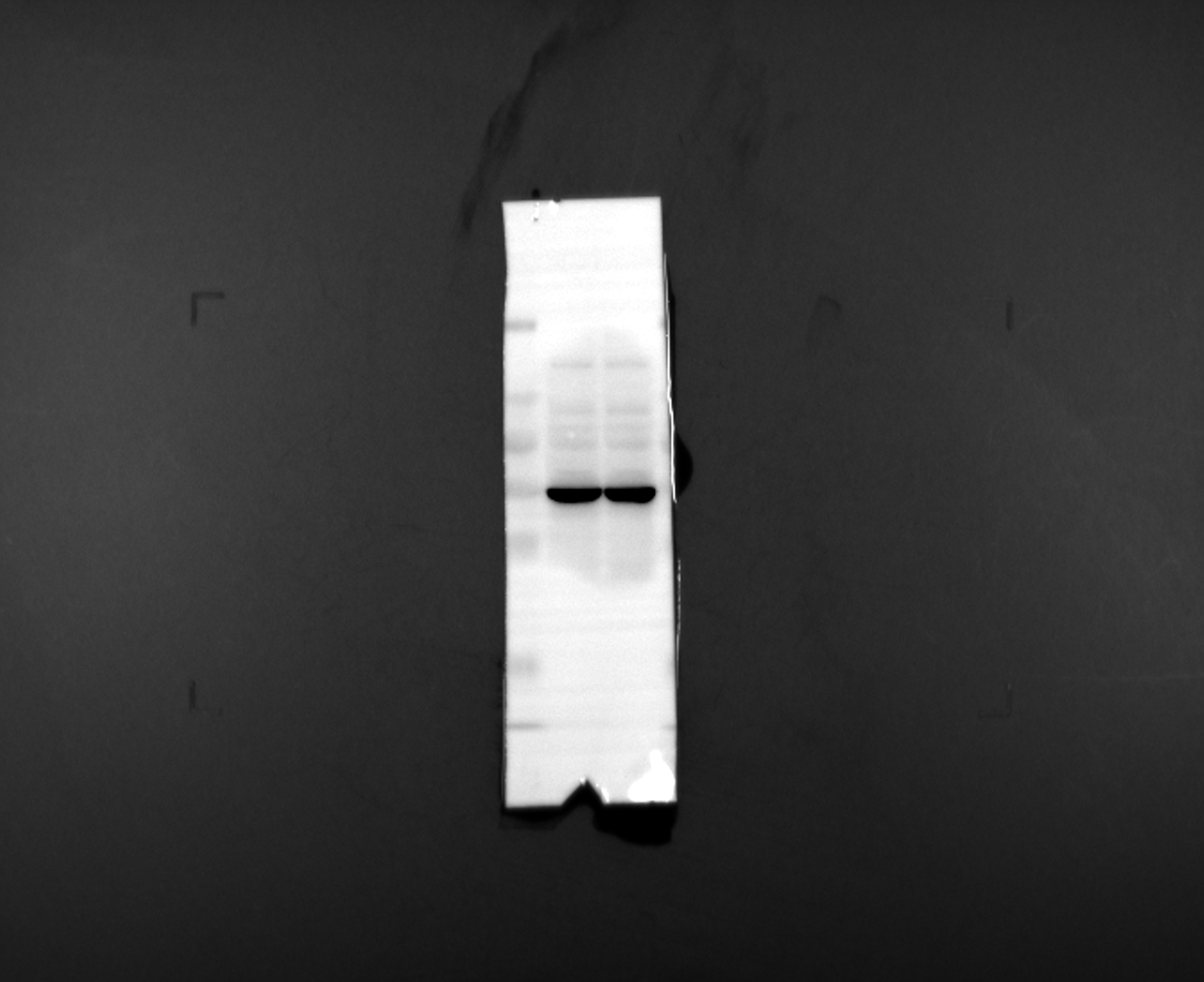


BAX


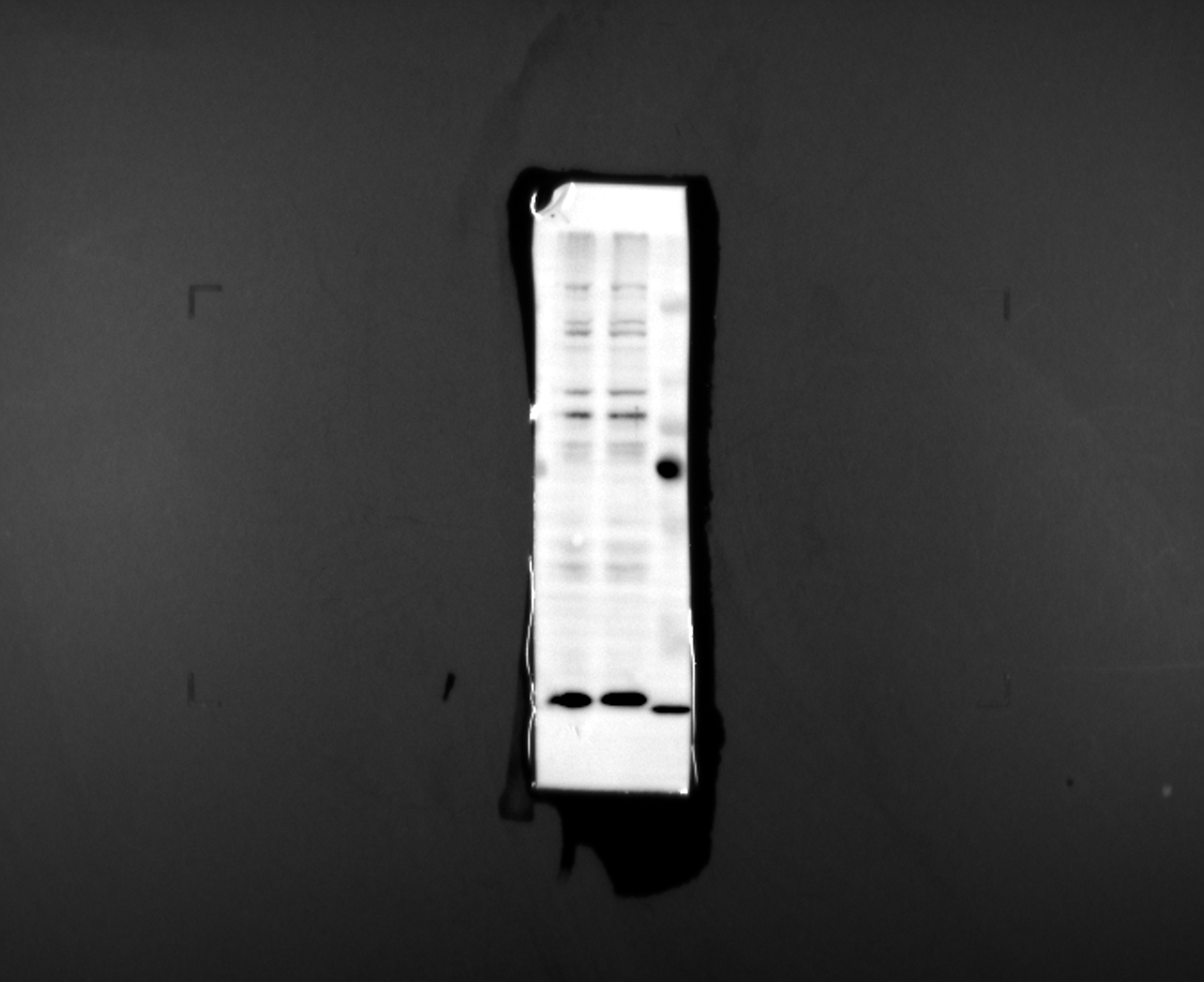


BCL2


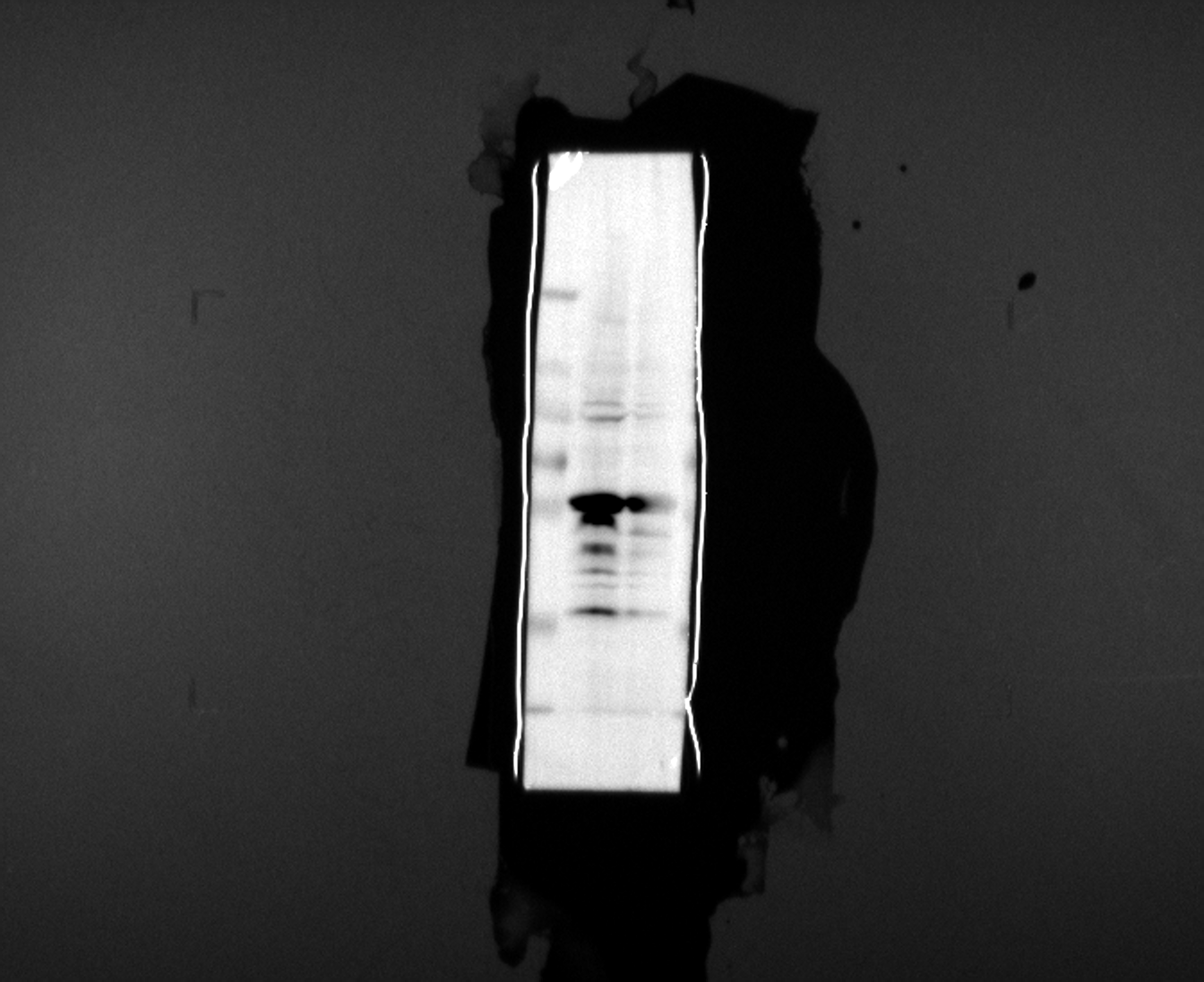


Caspase3


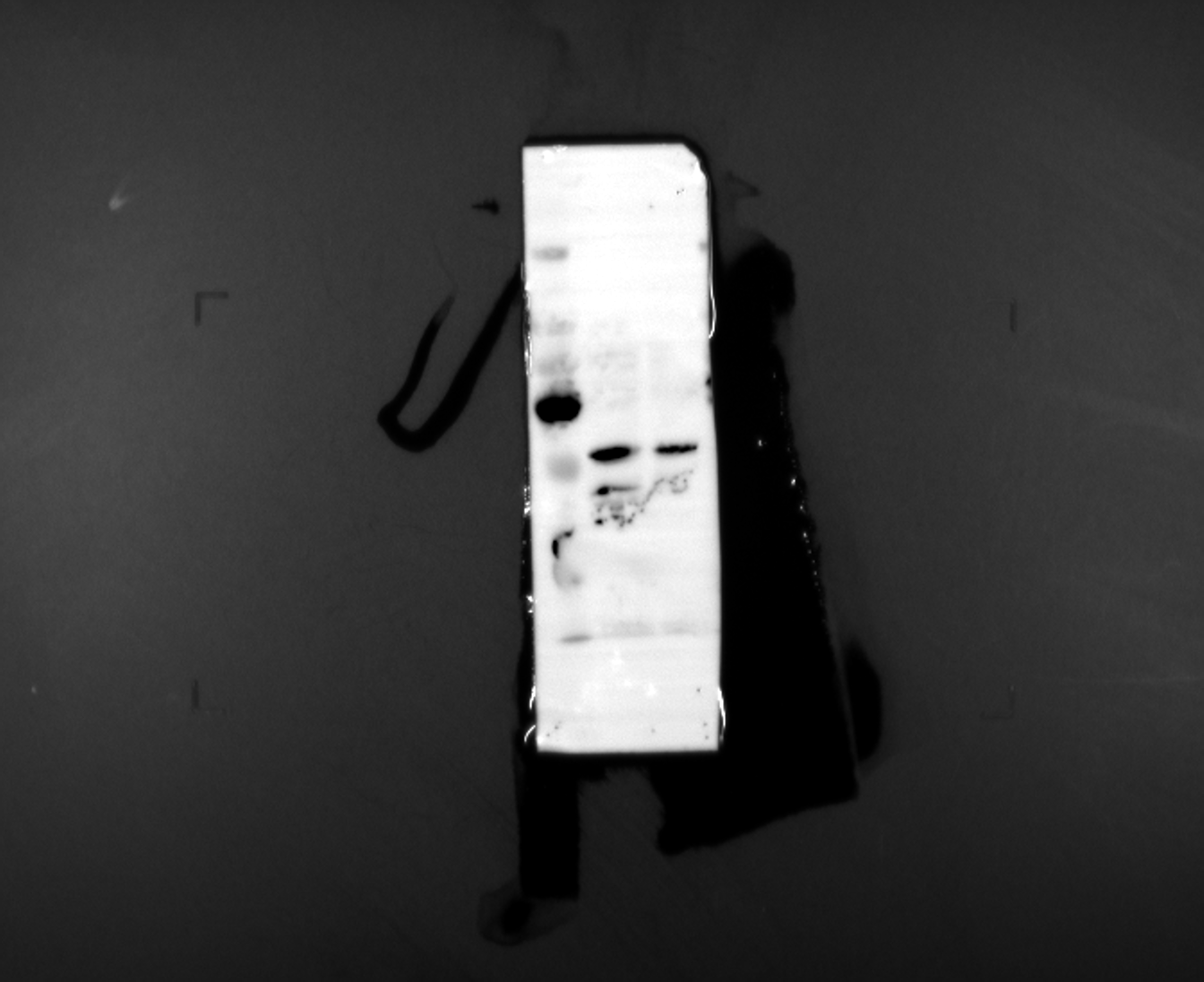


Cleved caspase3


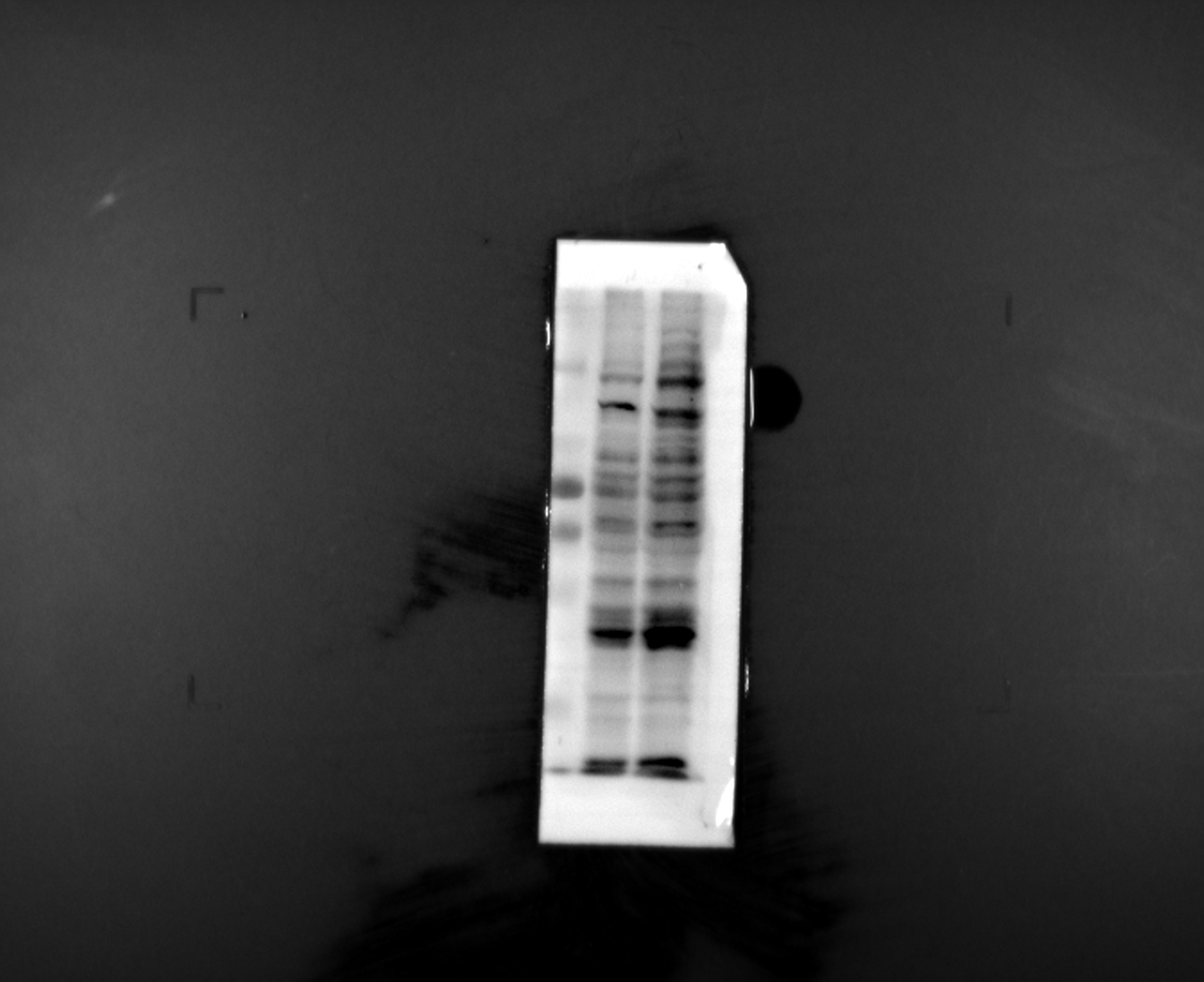


HuCCT-1

ACTIN


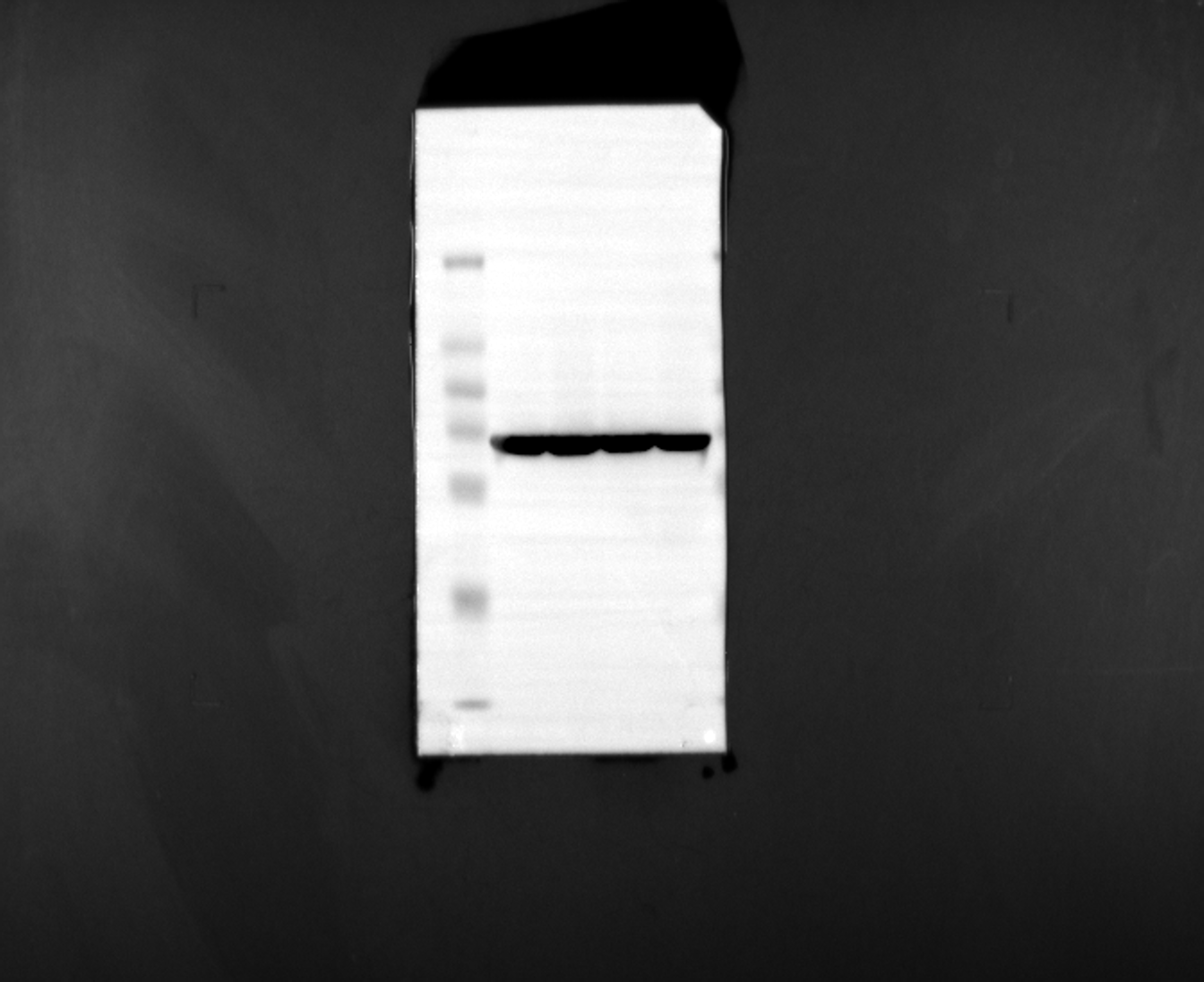


MTOR


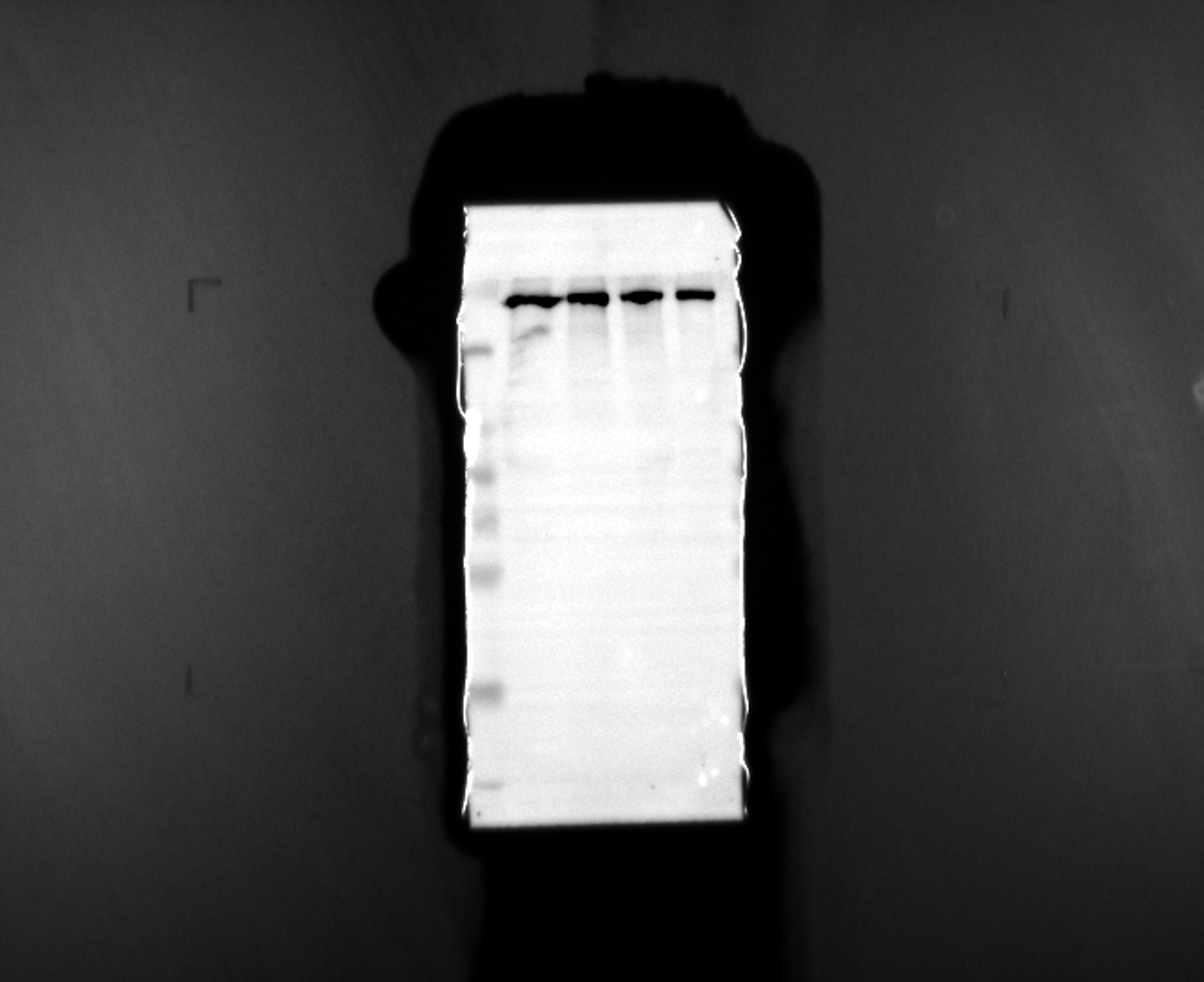


P-MTOR


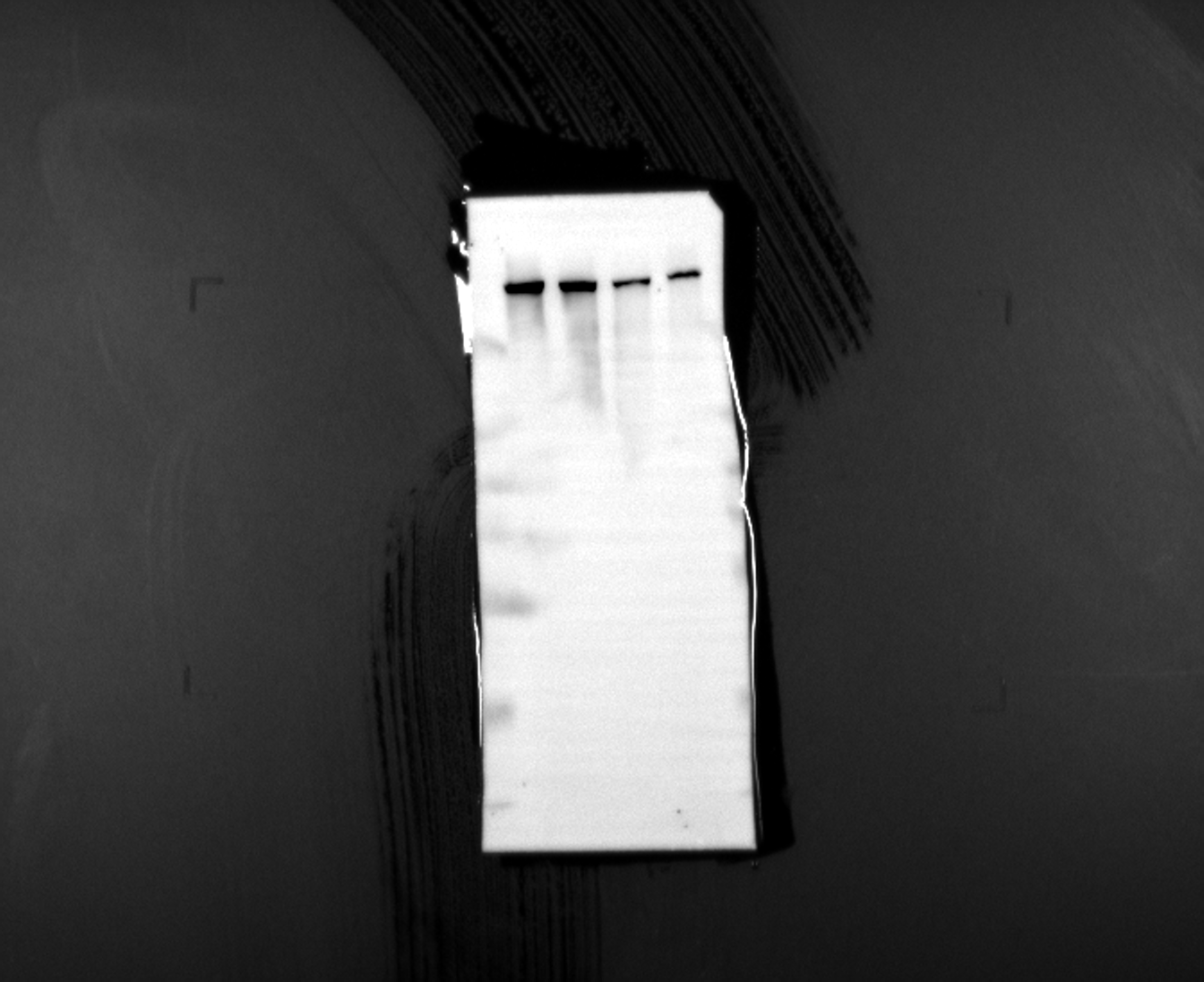


PI3K


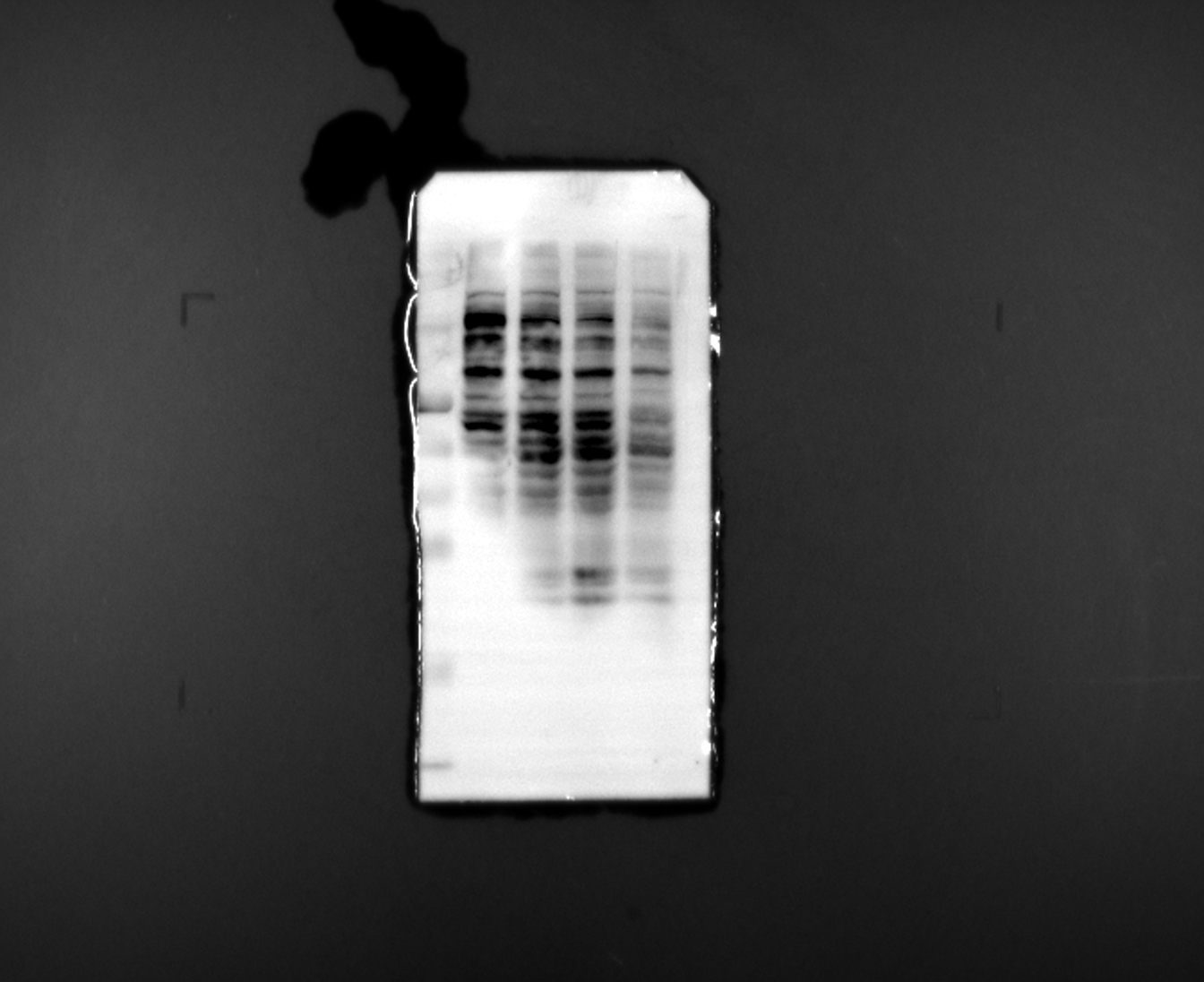


AKT


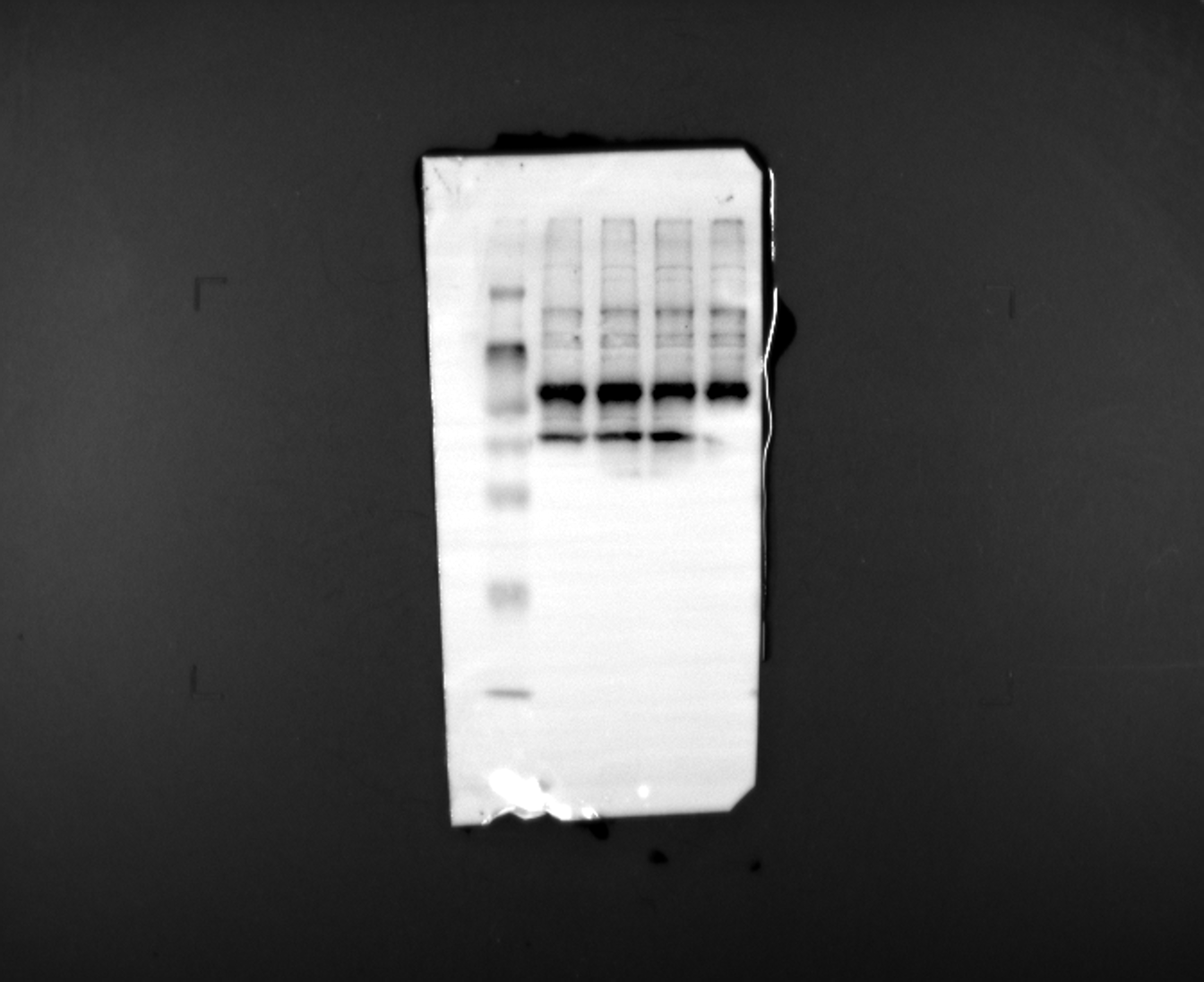


P-AKT


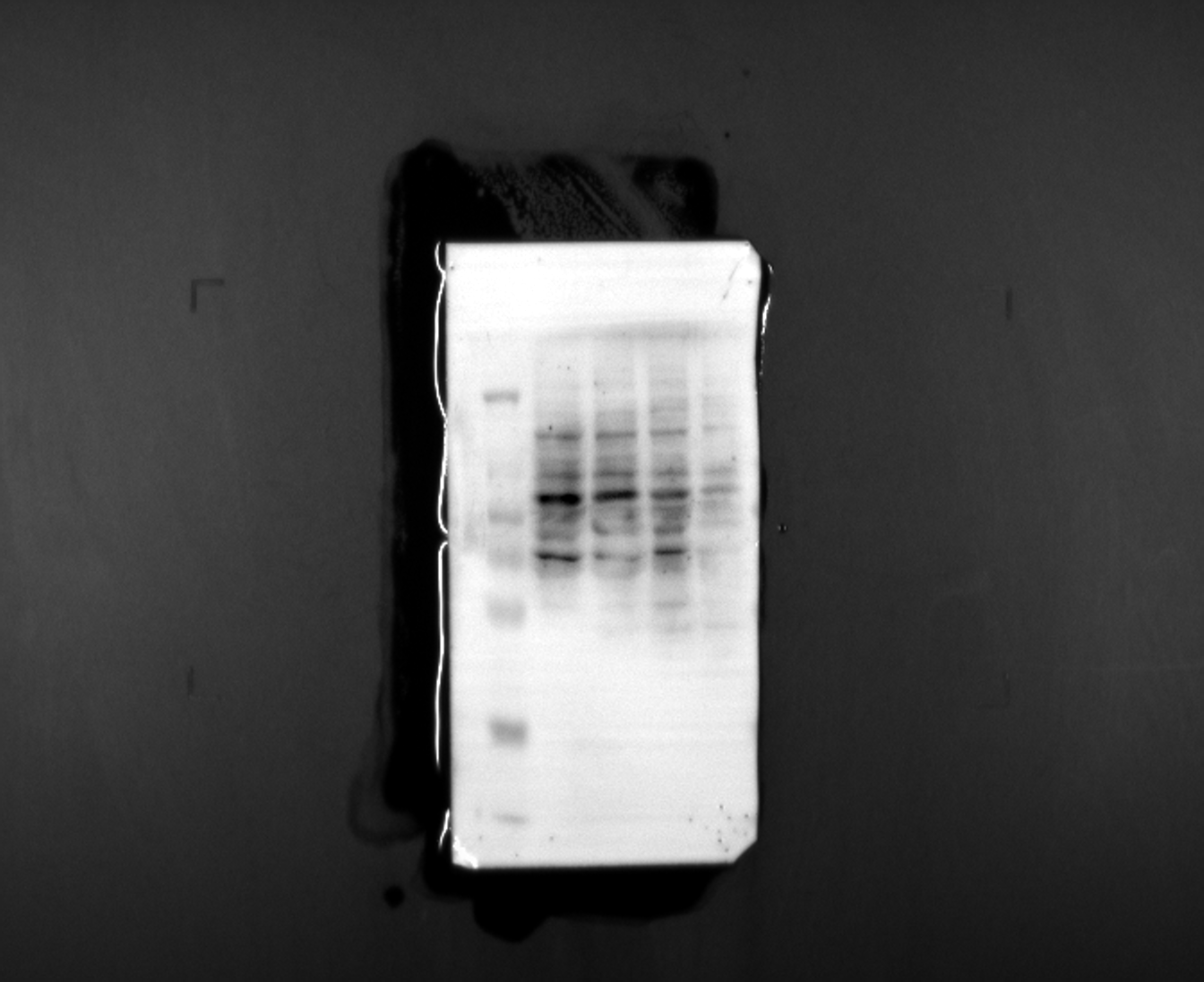


RBE

ACTIN


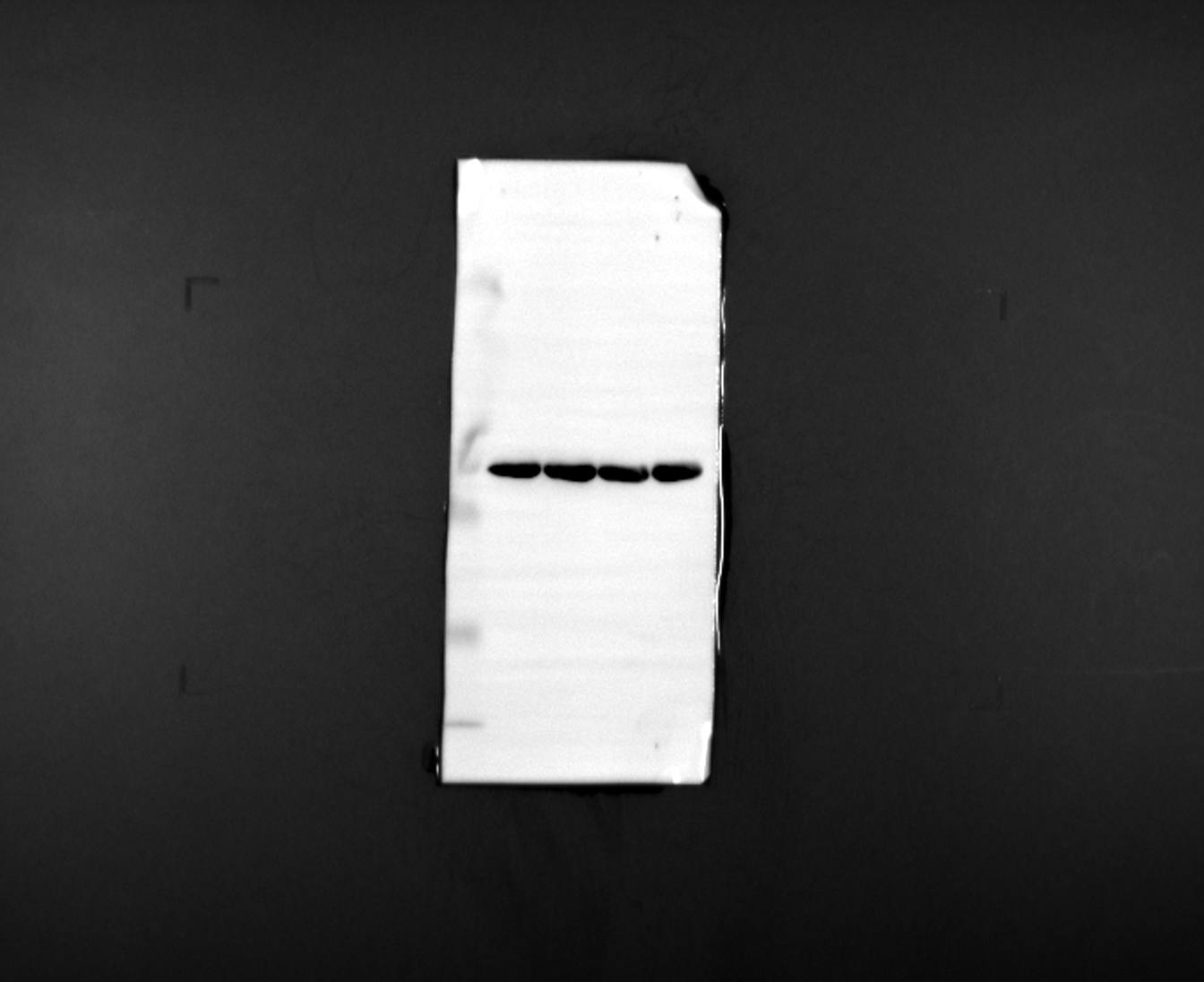


MTOR


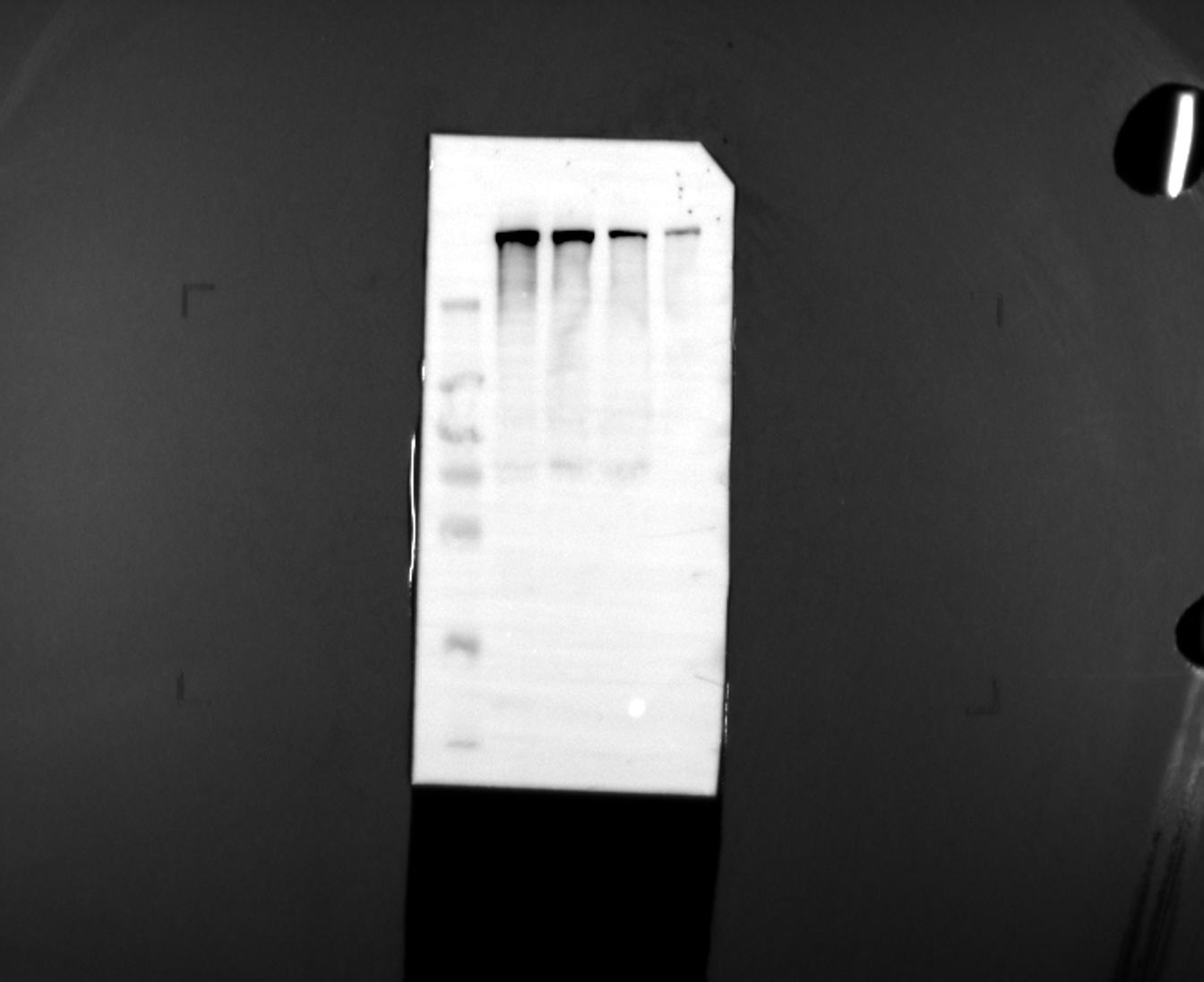


P-MTOR


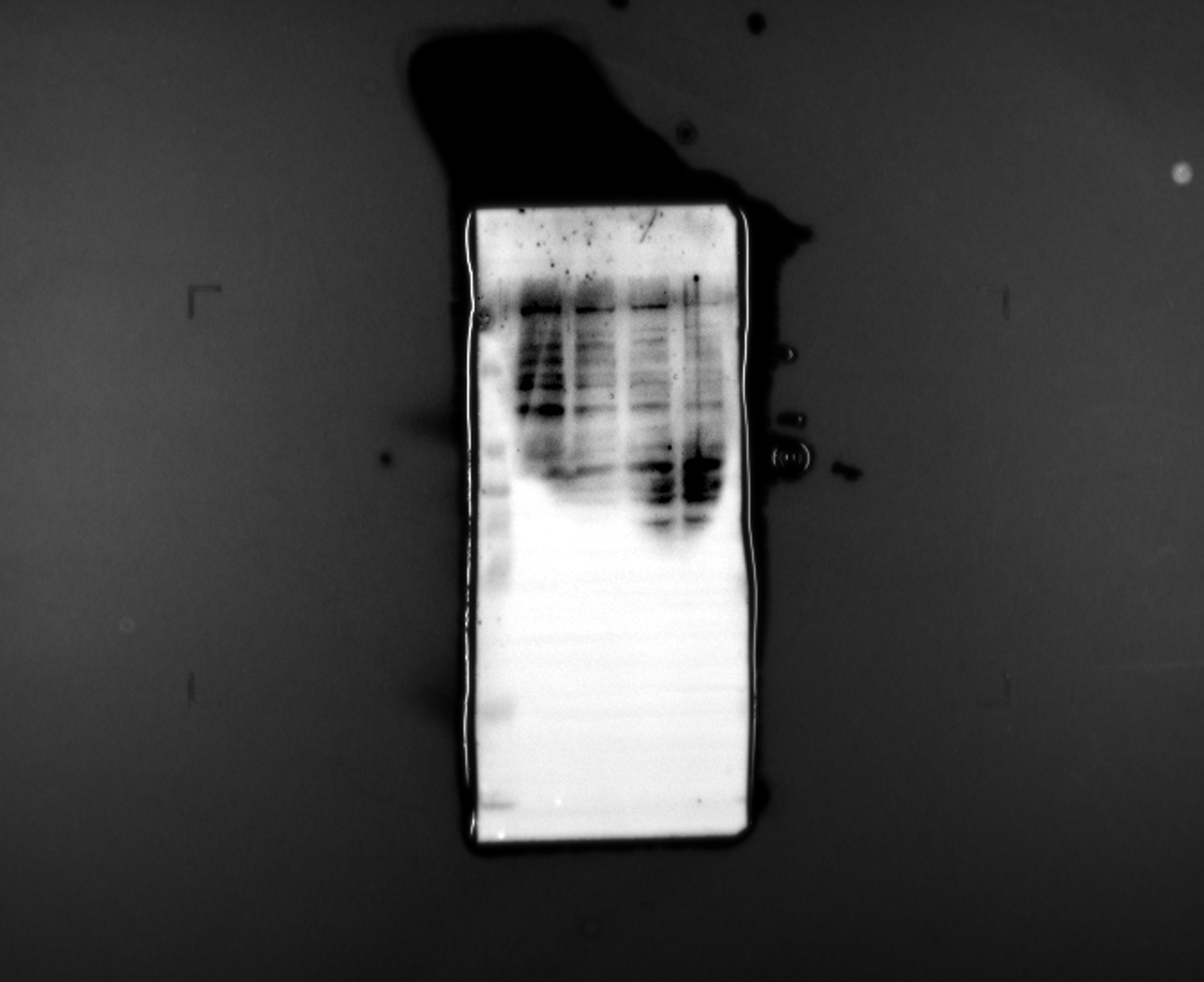


PI3K


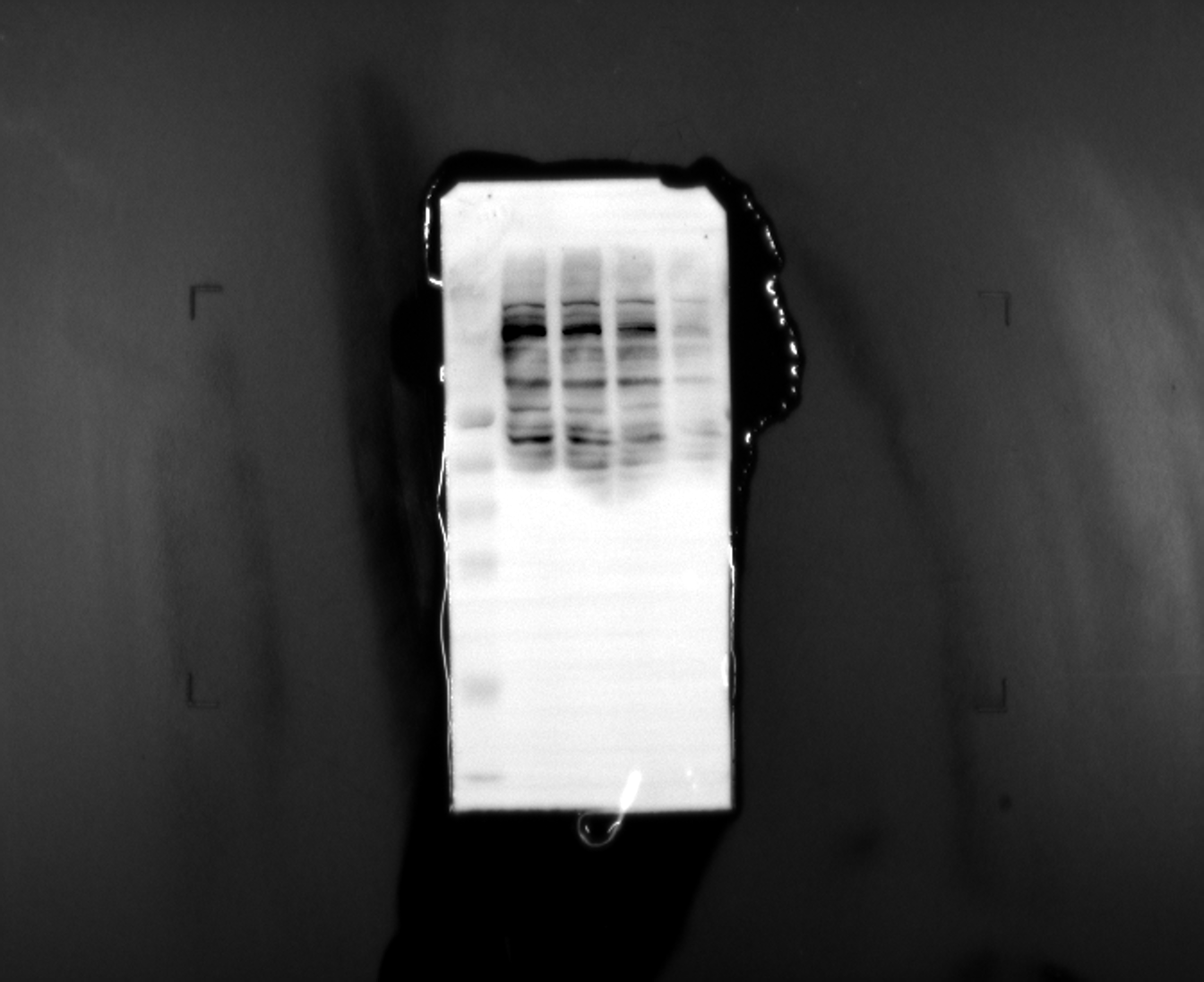


AKT


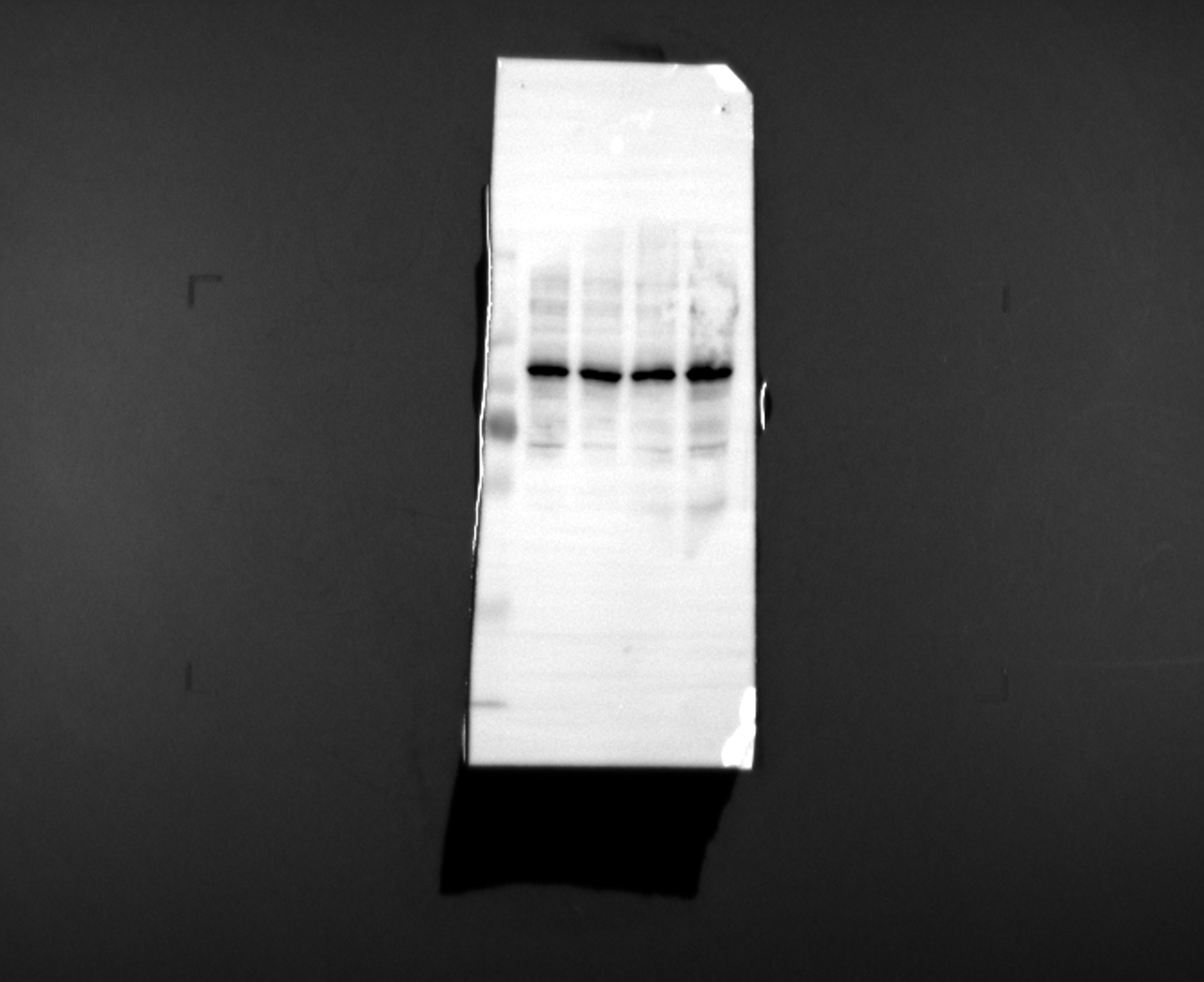


P-AKT


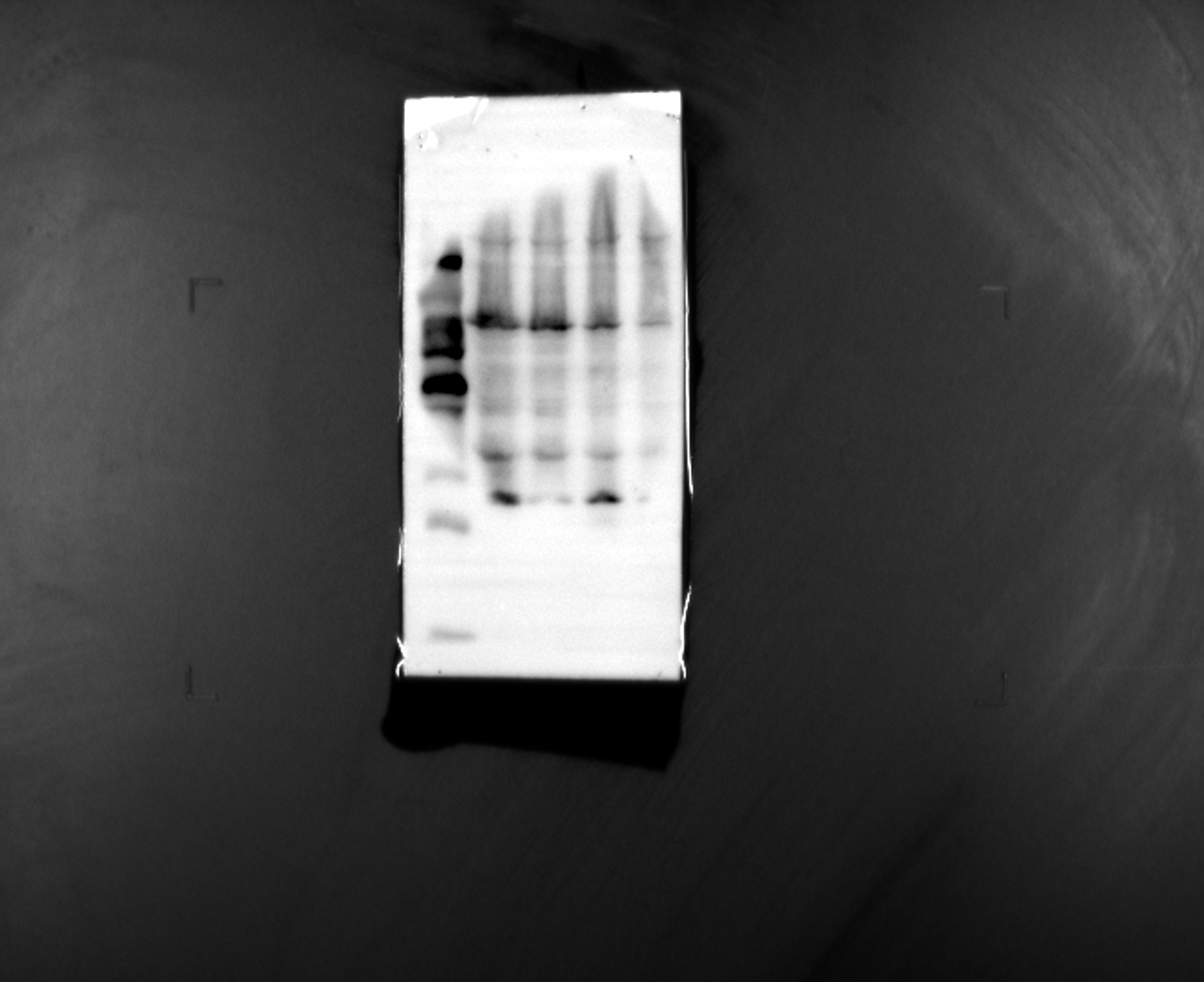


HuCCT-1

ACTIN


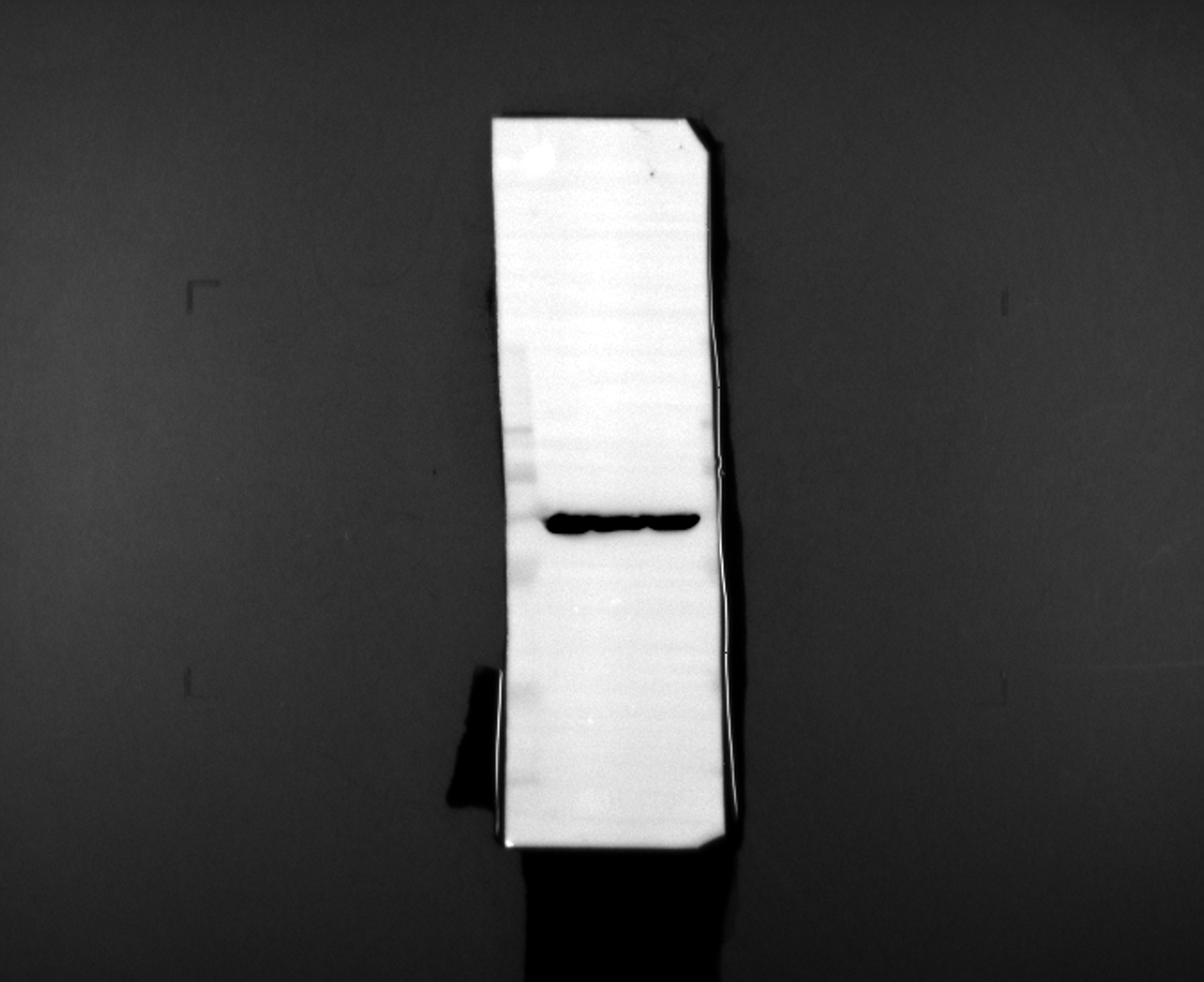


MTOR


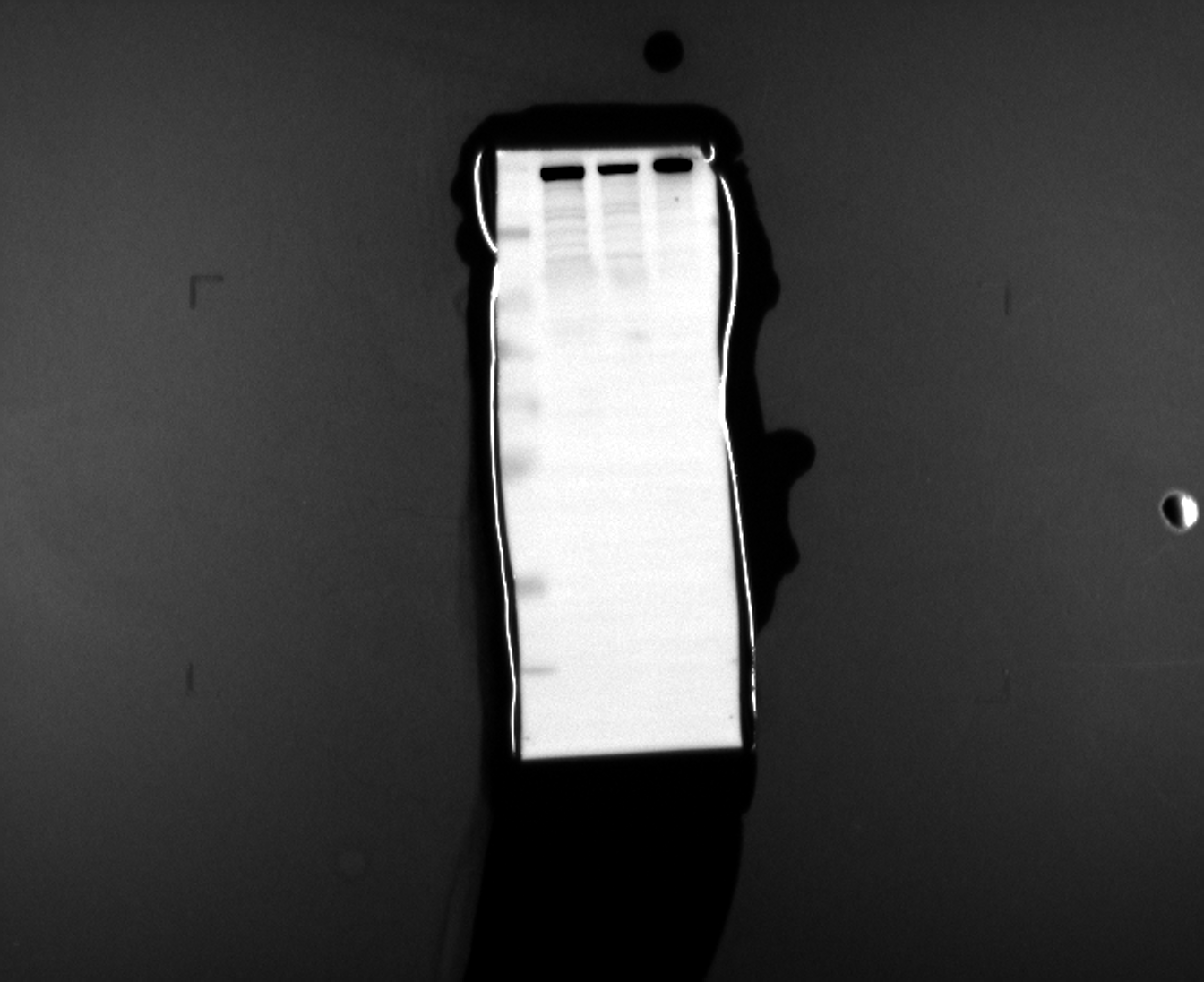


P-MTOR


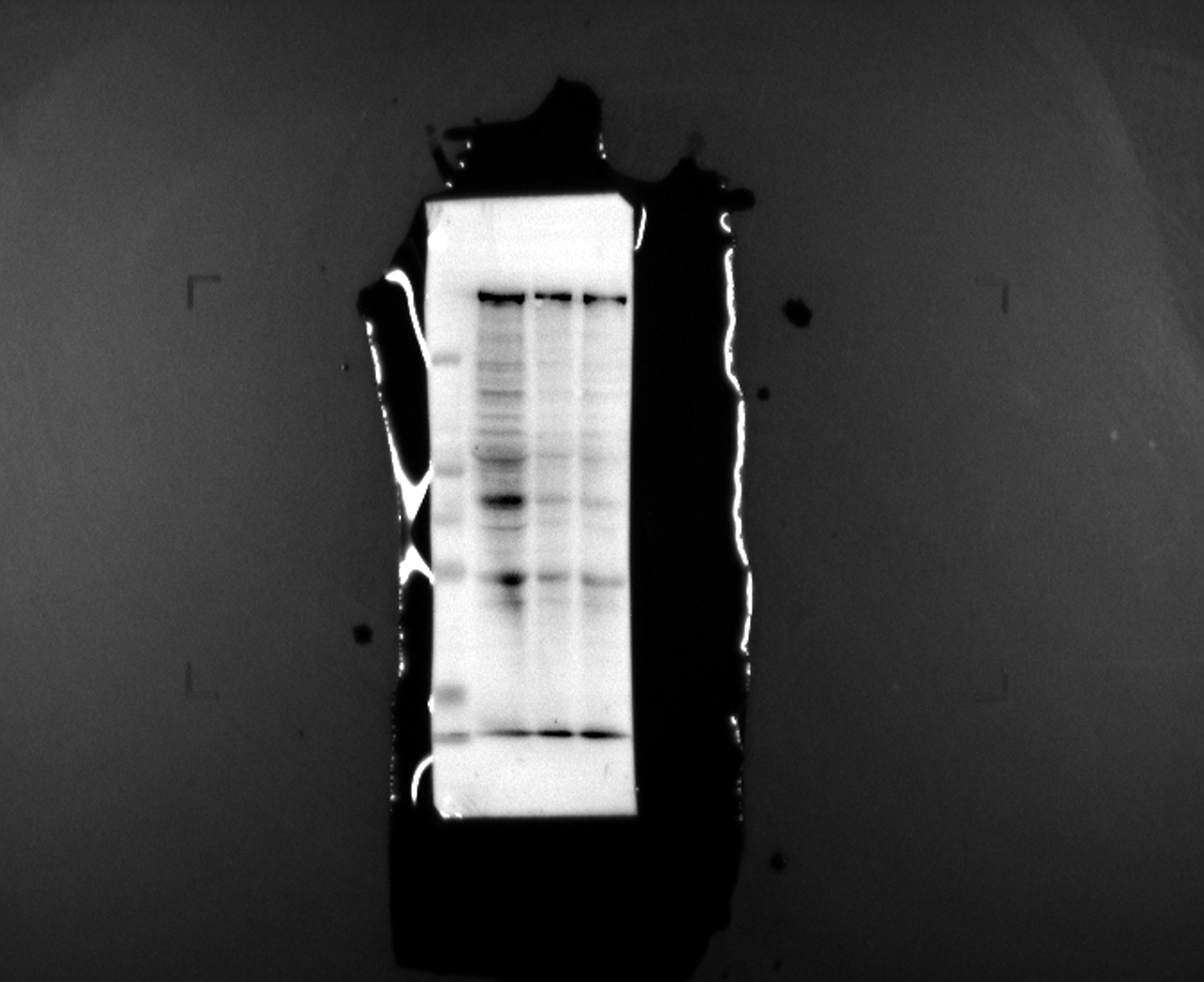


PI3K


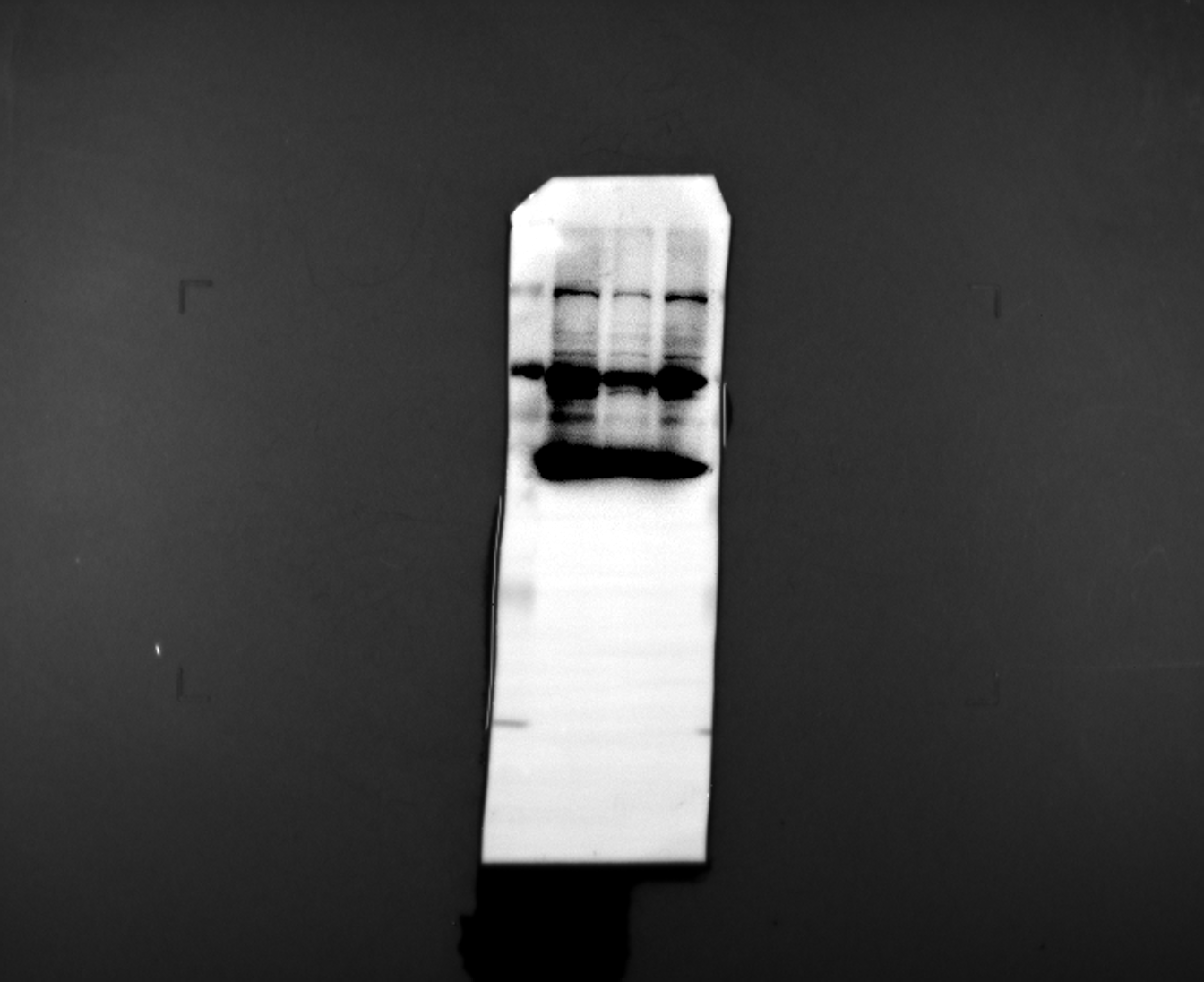


AKT


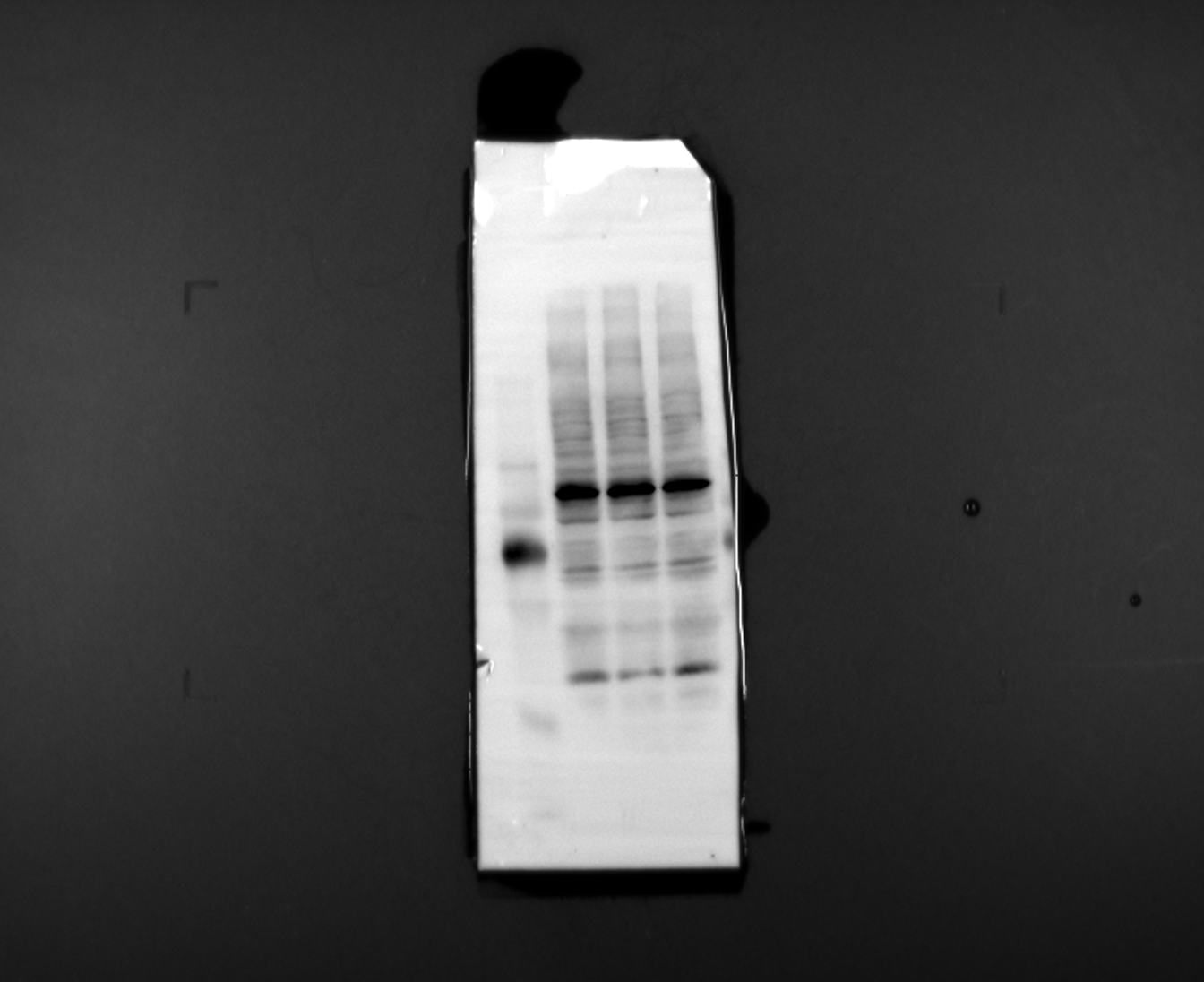


P-AKT


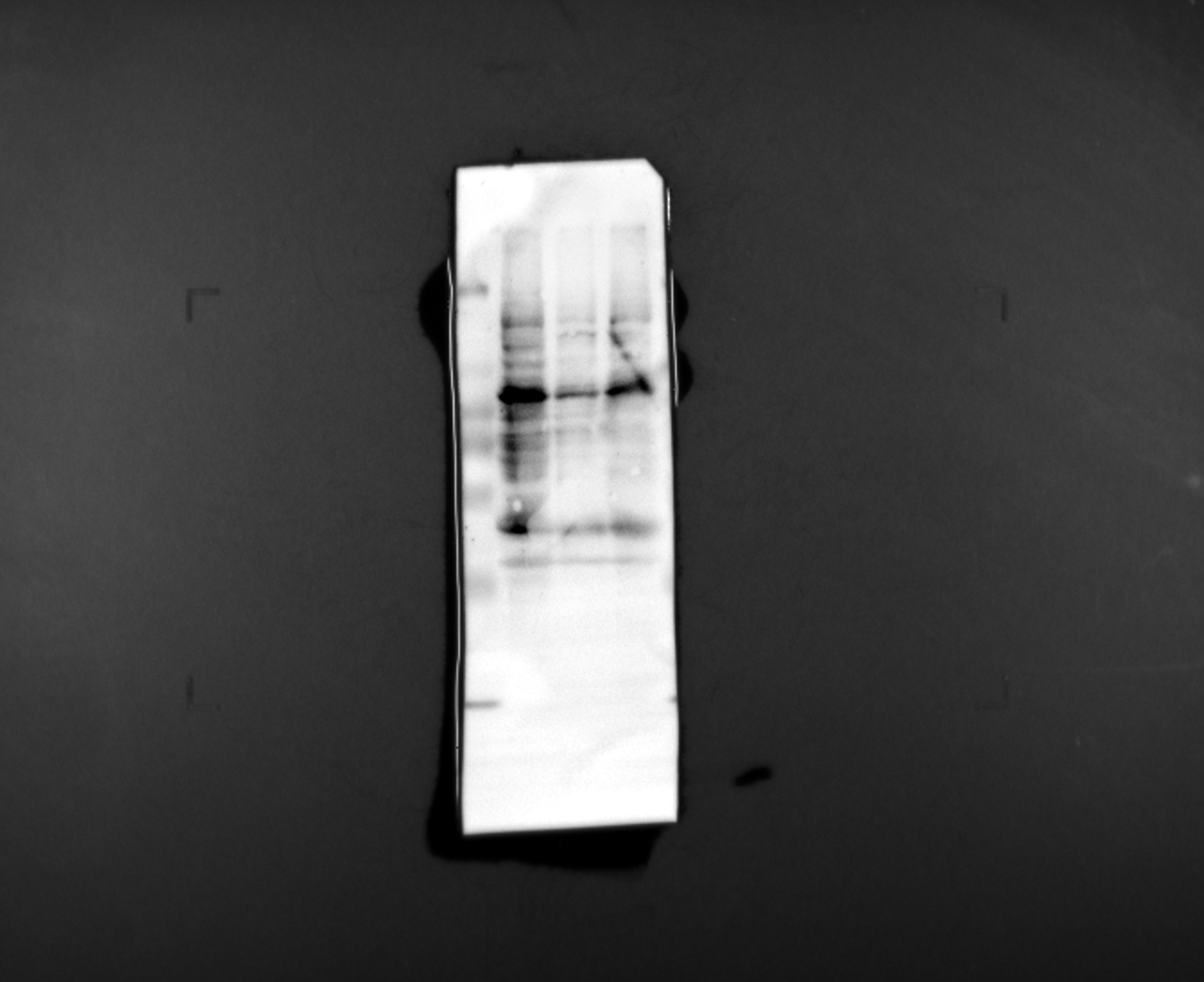


RBE

ACTIN


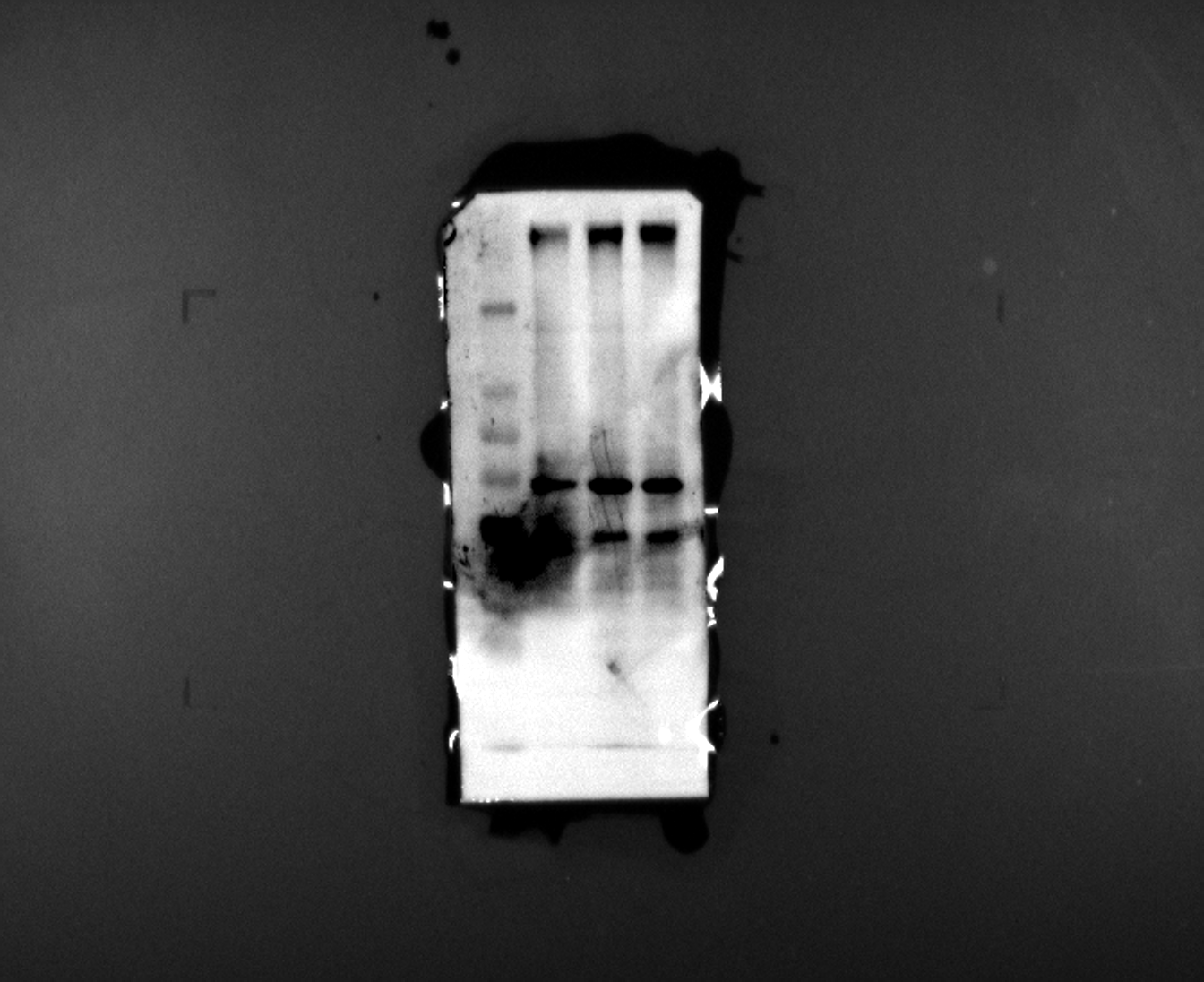


MTOR


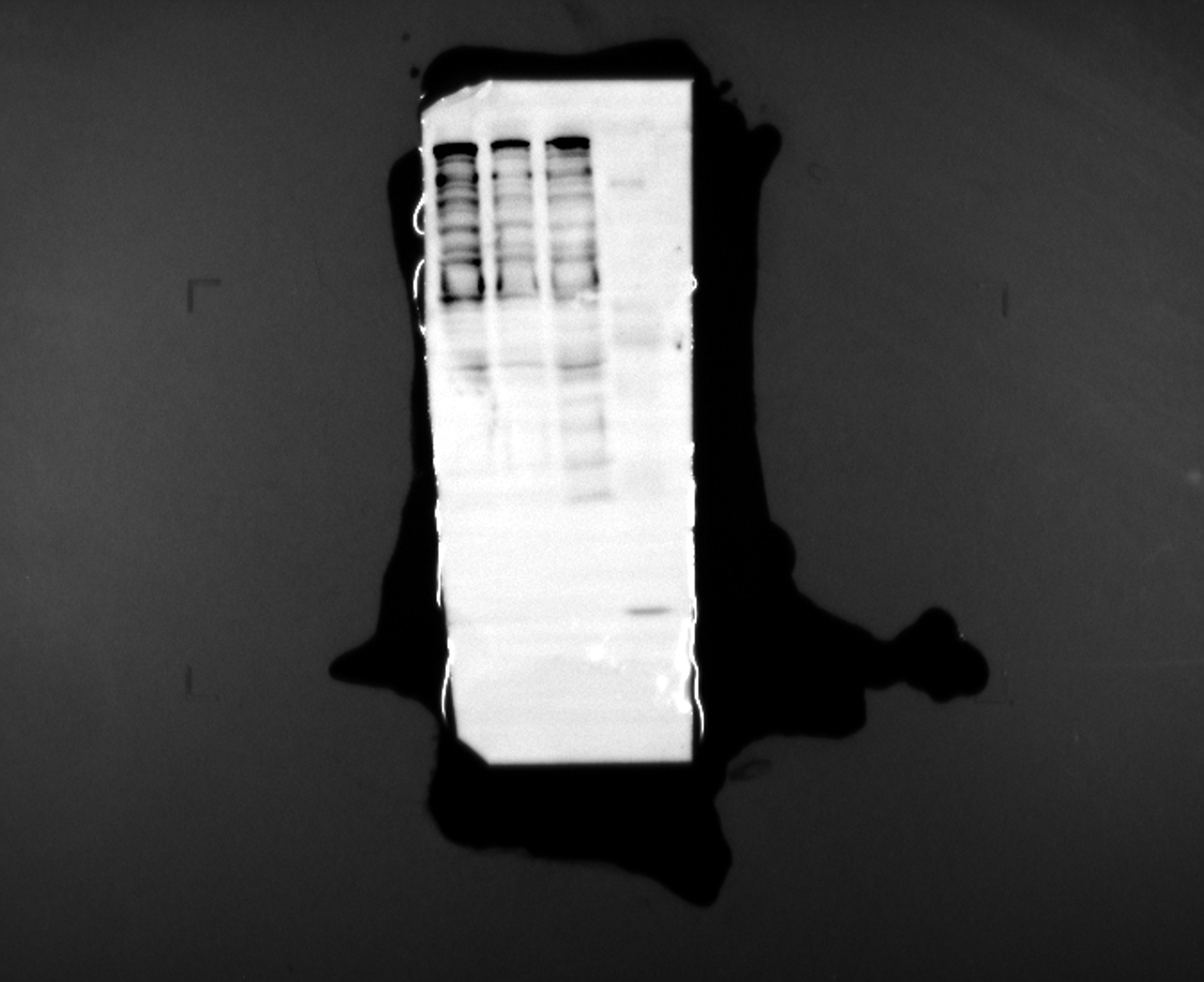


P-MTOR


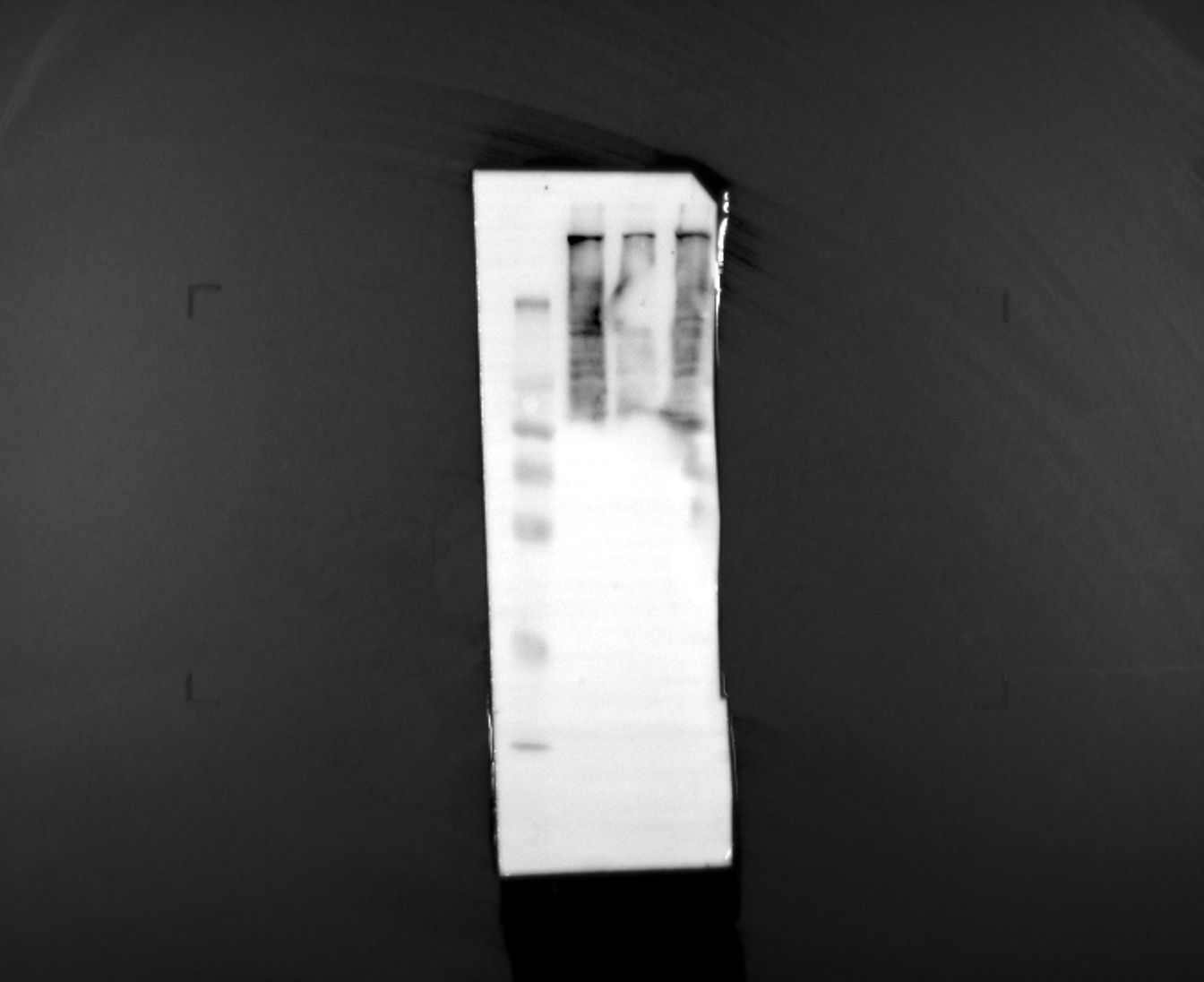


PI3K


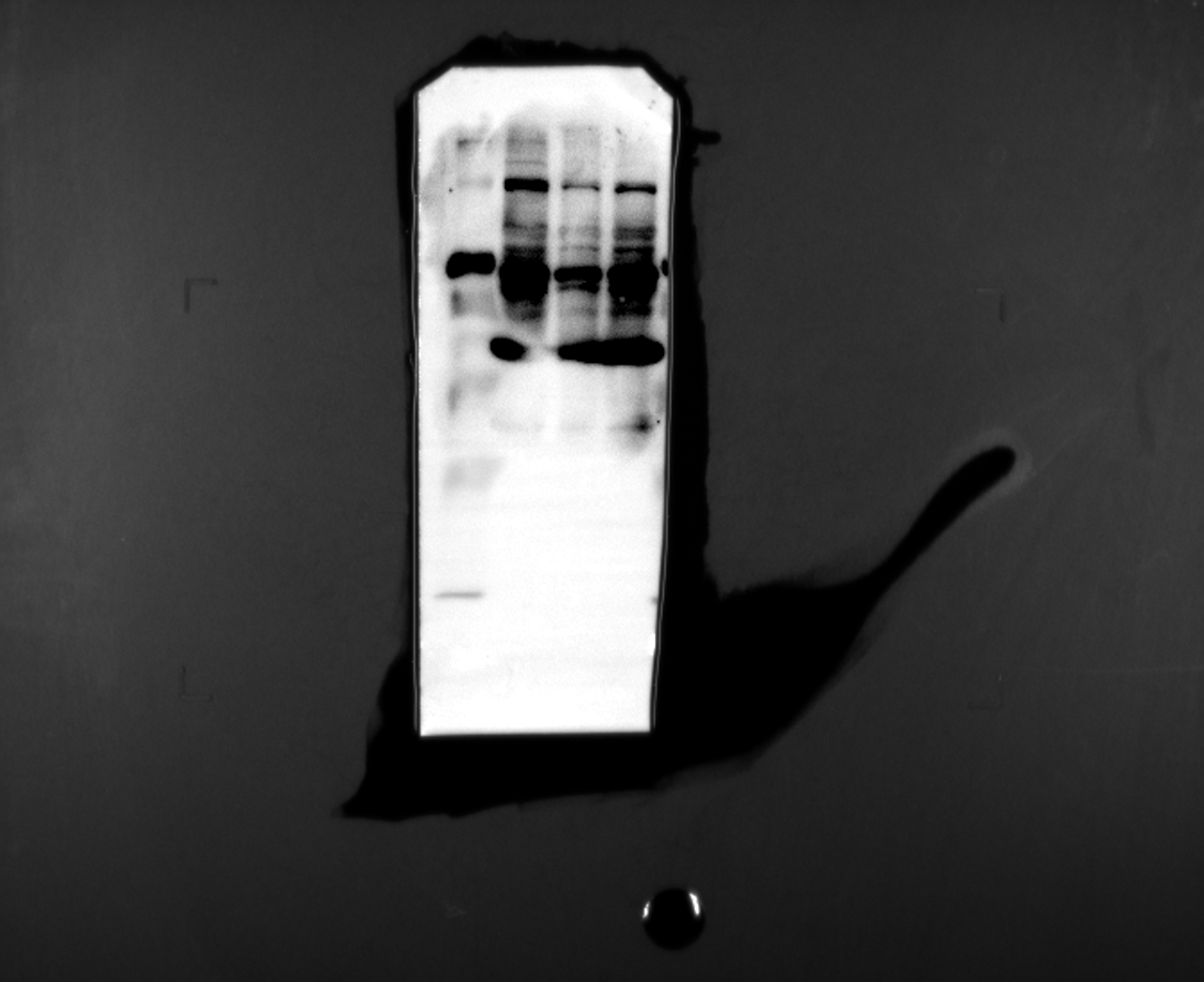


AKT


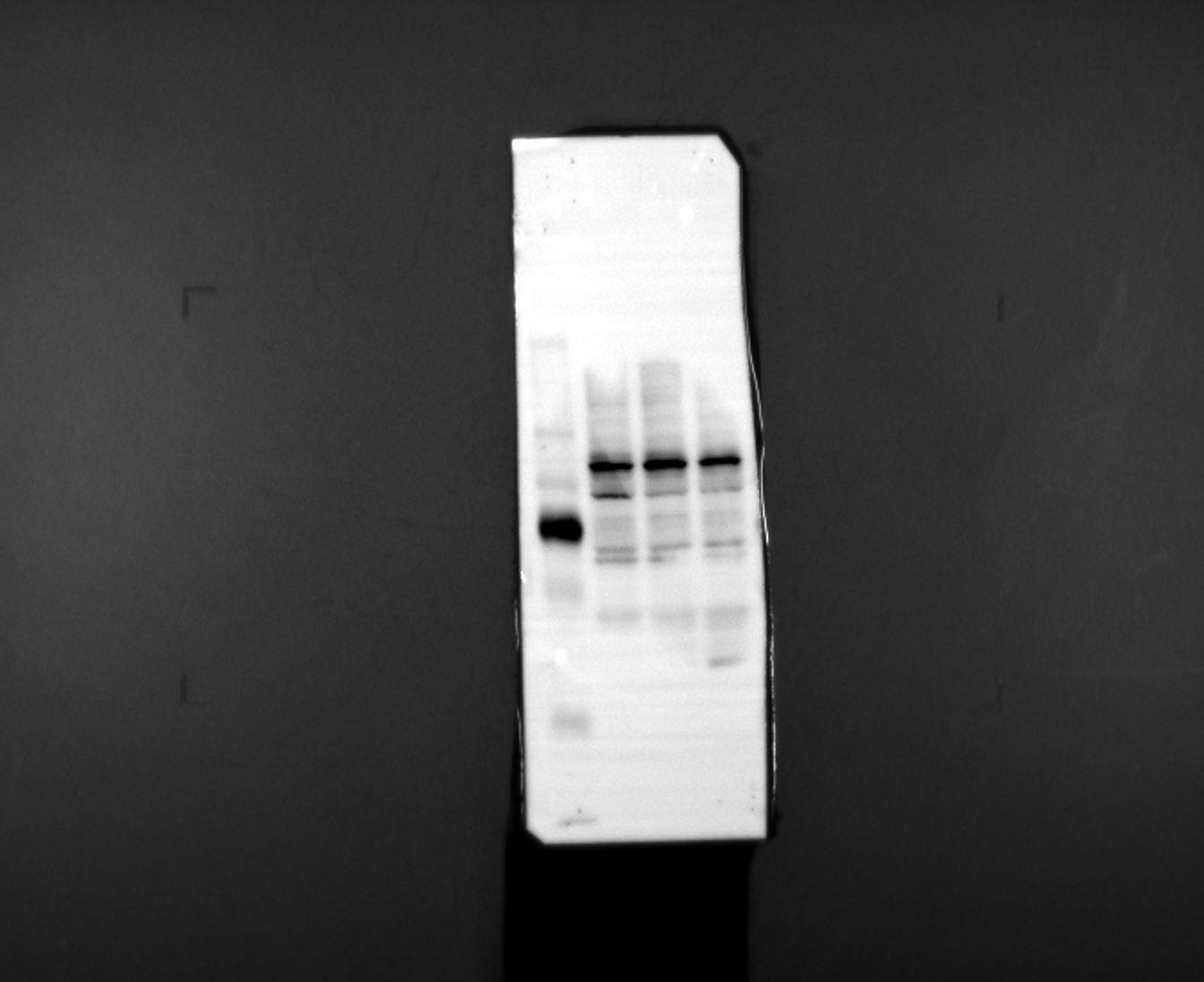


P-AKT


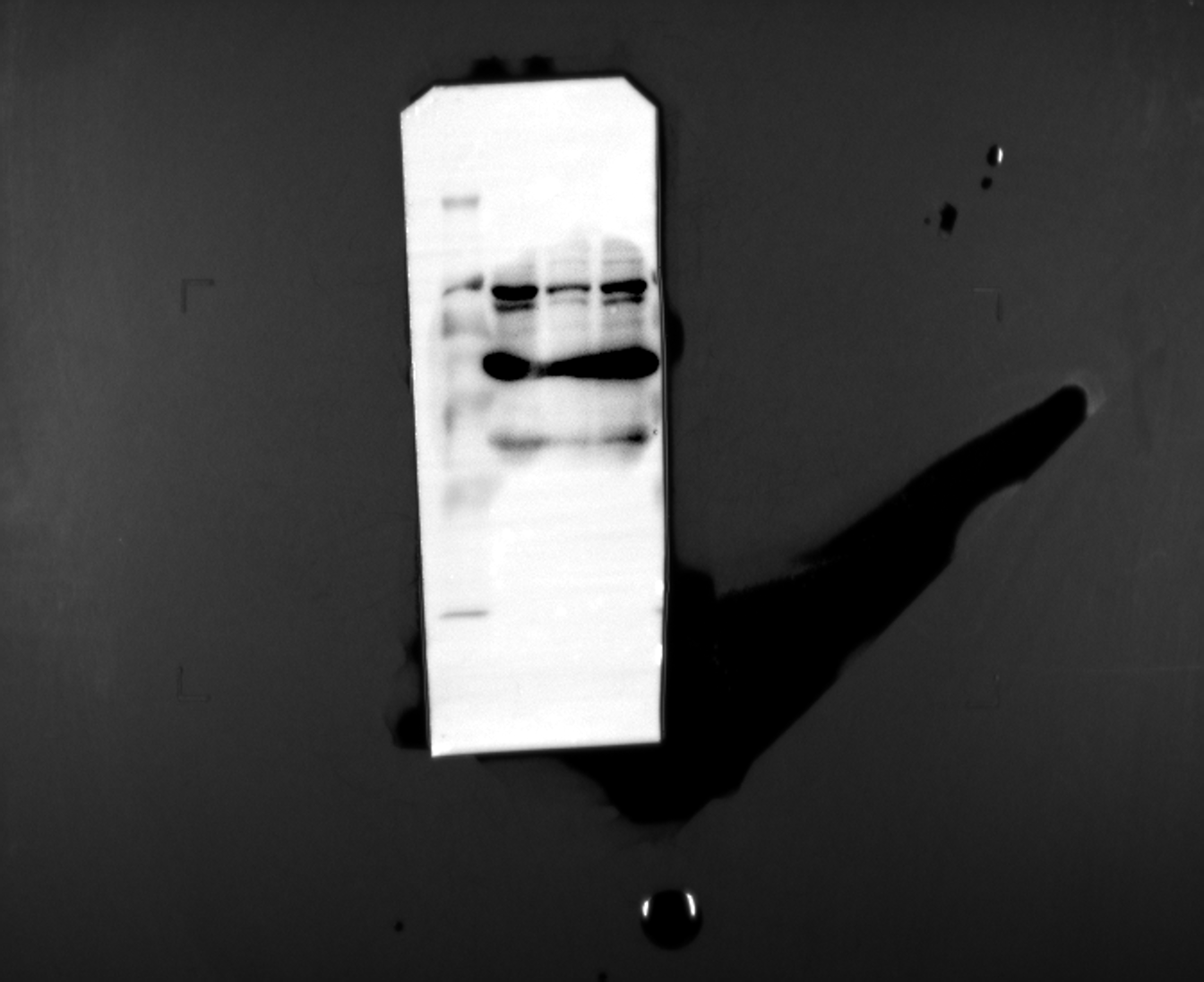


The second experiment

HUCCT-1

BCL-2





180KDa

140KDa

100KDa

75KDa

60KDa

45KDa

35KDa

25KDa

15KDa

BAX





Caspase-3





Cleaved-caspase-3





ACTIN





RBE

Cleaved-caspase-3





Caspase-3





BCL-2





BAX





ACTIN





HUCCT-1

PI3K p-Akt







MTOR Akt







ACTIN p-MTOR







RBE

PI3K p-Akt







MTOR Akt







ACTIN p-MTOR







HUCCT-1

PI3K p-Akt







ACTIN MTOR







Akt p-MTOR







RBE

Akt p-MTOR







PI3K p-Akt







ACTIN MTOR







The third experiment

HUCCT-1

Caspase-3 BCL-2







BAX ACTIN







Cleaved-caspase-3





RBE

Cleaved-caspase-3 Caspase-3

**



**

**BCL-2 BAX**

**



**

**ACTIN**

**

**

HUCCT-1

PI3K p-Akt







ACTIN MTOR







Akt p-MTOR







RBE

PI3K p-Akt







MTOR Akt







ACTIN p-MTOR







HUCCT-1

p-Akt MTOR







Akt ACTIN







p-MTOR PI3K







RBE

PI3K p-Akt







ACTIN MTOR







Akt p-MTOR
